# Supplementary material for: Effects of biofertilizers on the growth, leaf physiological indices and chlorophyll fluorescence response of spinach seedlings
Source: PLoS One. 2023 Dec 14;18(12):e0294349. doi: 10.1371/journal.pone.0294349 (PMC10721093; doi:10.1371/journal.pone.0294349)
Supplement: Attachment — Submitted filename: PONE-D-23-13632_R1_reviewer (1).pdf [file pone.0294349.s004.pdf]

PLOS ONE

Effects of biofertilizers on the growth, leaf physiological Indices and chlorophyll fluorescence response of spinach seedlings

--Manuscript Draft--

Manuscript Number:	PONE-D-23-13632R1
Article Type:	Research Article
Full Title:	Effects of biofertilizers on the growth, leaf physiological Indices and chlorophyll fluorescence response of spinach seedlings
Short Title:	Biofertilizers' effects on the growth of spinach
Corresponding Author:	Beibei Zhang<br>Pingdingshan University<br>Baoji, CHINA
Keywords:	Bacillus; Growth; Physiological characters; Chlorophyll fluorescence
Abstract:	Chemcial fertilizer as the main strategy for improving the vegetable yields was excessively applied in recent years which led to progressively serious soil problems such as the soil acidification. According the situation, five different biofertilizer treatments [no fertilizer (CK), inoculations of Bacillus subtilis (Bs, T1), combination of Bs and Bacillus mucilaginosus (Bs+Bm, T2), Bs and Bacillus amyloliquefaciens (Bs+Ba, T3), and Bm+Ba (T4)] were conducted to investigate the effect of the growth, leaf physiological indices, and chlorophyll fluorescence of spinach seedlings in the growth chamber. The growth and physiological indices of the spinach seedlings attained a maximum under the T2 treatments. Under the T2 treatment, the ABS/RC (Absorption flux per RC), TR0/RC (Trapping flux per RC), and ET0/RC (Electron transport flux per RC) was significantly increased, while the DI0/RC [Dissipated energy flux per RC (at t=0)] was decreased. The OJIP curve was improved under of the inoculations of fertilizers, and the increasing range was the largest under the T2 treatment. The leaf light response curve (LC) was also significantly increased under the T2 treatment. The plant growth characteristics [leaf length (LL), leaf weight (LW), plant height (PH)] were positively correlated with the J-I-P test chlorophyll fluorescence parameters [PIABS (Performance index for energy conservation from exciton to the reduction of intersystem electron acceptors), φP0 (Maximum quantum yield of primary photochemistry), φE0 (Quantum yield of electron transport), ψ0 (The probability that a trapped exciton moved an electron in electron transport chain further than QA-), TR0/RC, and ET0/RC] while negatively correlated with φD0 (Quantum yield of energy dissipation) and DI0/RC. The leaf physiological characteristics [SP (soluble protein concentrations), SC (soluble carbohydrate concentrations), Chl a (chlorophyll a), Chl b (chlorophyll b), Chl a+b, Chl a/b, and WP (water potential)] were positively correlated with the J-I-P test chlorophyll fluorescence parameters (PIABS, φP0, φE0, ψ0, ABS/RC, TR0/RC, and ET0/RC) while negatively correlated with φD0 and DI0/RC. These results indicated that the combination of Bs+Bm inoculations promoted the growth of the spinach and improved the adaptability of the vegetable to acid soil while Ba inoculation didn’t have any effects to plants.
Order of Authors:	Beibei Zhang
	Hui Zhang
	Di Lu
	Liping Cheng
	Jiajia Li
Response to Reviewers:	Response to reviewers’ comments:<br><br>Re: Manuscript # PONE-D-23-13632:<br>Review Comments to the Author<br><br>Reviewer #1: The authors have fulfilled the requirements for the manuscript, presented the research data, and the conclusions are substantiated.

Powered by Editorial Manager® and ProduXion Manager® from Aries Systems Corporation

The question - if there is an effect of fertilizers on chlorophyll fluorescence, then why is there no significant effect on the amount of chlorophyll? Then what is the reason for the change in fluorescence, it only with morphological changes in the leaf?

-Accepted and Revised. In ms, the mean values were compared, and the difference in the amount of chlorophyll was also significant, but the F value was written incorrectly, we are so sorry about the mistake. When facing the external changes, the first change is the chlorophyll fluorescence, which could change the chlorophyll concentrations of the leaves and ultimately affects the morphological changes in the leaf.

Reviewer #2: This was very well thought out experiment to test the effects of different inoculants on early spinach growth.

I had some issues reading and understanding parts of the manuscript. An additional edit for clarity would make it easier for readers to understand the work done and discussion presented. For example in the first paragraph of the discussion lines 246-250 “In this study, the inoculations of biofertilizer effectively improved the plant height, leaf length, and leaf weight of spinach, among which the growth status of plants with the inoculations of Bs and Bm was more dominant while which was similar to the results of Samia et al. [34] while the inoculation of Ba didn’t have more effects.”. Breaking long sentences like this up would improve how well this information is presented to the readers. Similarly changing awkward wording like “while which” and “didn’t have more effects” would provide a clearer point to the discussion. Improving sections like this throughout the manuscript would greatly improve the manuscript.

-Accepted and Revised. We have revised in the ms.

To also improve clarity, I would recommend that a more consistent representation of the treatments be decided upon. In parts of the manuscript the treatments are referred to as Bs/T1 or Bm/T2. For instance, in the introduction lines 129-131 identify the treatments as multiple identifiers (Bs, T1 or Bm T2), in the conclusion on line 328 the individual inoculants are identified (Bs and Bm), Fig 1 G has the graphs as (B,C,D,E, and F), , Table 2 lists the treatments as CK,T1,T2,T3,and T4. Deciding on a consistent representation for the treatments for the figures, tables, and text will make it easier for readers to better follow the effects of the treatments.

-Accepted and Revised. Thank you for you comments, we have revised the representation making the ms more consistent and better understanding. In the lines 129-131, that was the material introductions. For the treatment introduce, it was on the experiment design part.

In addition to the editing, I would recommend additional discussion of the impacts that the inoculants had on plant nitrogen status. It was mentioned in the introduction on lines 81-82 the impact that Bs has on changing the availability of nitrogen and phosphorus in the soil. Nitrogen was never mentioned again in the manuscript. Nitrogen is a major plant nutrient. Despite nitrogen status not being directly measured total chlorophyll could be used as a surrogate for plant nitrogen status. The impact the inoculants had on nitrogen would be a much better claim than the impact increased phosphorous had on ATP and NADPH production as was made in lines 264-268. With the data presented in table 3 the relationships between the various inoculants become much more apparent when look at the chlorophyll a+b data. When Bs and Bm were inoculated together (T2) the plants had more chlorophyll and likely nitrogen and phosphorous. Whereas when Bs or Bm were paired with Ba there was less chlorophyll and likely nitrogen and phosphorous. Then the impacts on growth, leaf physiological indices and chlorophyll fluorescence response can be better framed around how the inoculant was impacting the plants. Presenting the results in the discussion in this context could better emphasize the potential impacts these inoculants have on early spinach growth and the importance or emphasis of species-specific inoculant and crop pairings.

-Accepted and Revised. Thank you for your comments; we have discussed more in the ms.

Powered by Editorial Manager® and ProduXion Manager® from Aries Systems Corporation

	Lastly, I recommend that something be added to the manuscript that better frames the results obtained from an experiment growing spinach in a growth chamber at only 300 μmol/(m2•s). Spinach can be grown in full sunlight, 2000 μmol m-2•s-1, and discussing the impacts that the treatments in a growth chamber study with such low light on PS2 antenna could be misleading. Shade and low light treatments will inherently lead to increased PS2 antenna length. Therefore, given the experiment conducted, it would be difficult to conclude or suggest that the treatments impacted antenna length. They could have contributed to antenna length but cannot be certain of their impact.<br><br>-Accepted and Revised. Thank you for your comments; we have discussed more in the discussion part. For the future experiment, we will design more light treatments to analyze the impact to spinach leaves.<br><br>Overall, the manuscript in its current form is hard to understand (which took me time to read through it again and again). Thus, I strongly suggest for a language editor to go through the paper and present the data and findings in a more comprehensible yet intelligible form of standard English. The title needs to be drastically changed to a more compelling statement as it appears to be too “amateur with preliminary data”. Although when you read through the paper, it actually contains noteworthy data that would make a significant contribution to the field of agriculture. The experiments and other analysis were indeed performed to high technical standard although the statistical analysis needs to be changed from Tukey’s test to Bonferroni test which is more appropriate based on its experimental design.<br><br>-Accepted and Revised. We let a professional English speaker reviewed the paper and according to his comments we checked the entire article and revised.
**Additional Information:**	
**Question**	**Response**
**Financial Disclosure**<br><br>Enter a financial disclosure statement that describes the sources of funding for the work included in this submission. Review the submission guidelines for detailed requirements. View published research articles from *PLOS ONE* for specific examples.<br><br>This statement is required for submission and **will appear in the published article** if the submission is accepted. Please make sure it is accurate.	This work was supported by the National Natural Science Foundation of China (NSFC, No. 41601016, 41771036), Special Support Program for High-Level Personnel Recruitment (Youth Top-Talent) of Shaanxi province, National Natural Science Foundation of Shaanxi province (No. 2015JM4136), Doctoral research start-up fee of Pingdingshan University (PXY-BSQD-2022039), Key projects of Shaanxi Provincial Department of Education(20JS011).<br><br>The funders had no role in study design, data collection and analysis, decision to publish, or preparation of the manuscript.

**Unfunded studies**<br>Enter: *The author(s) received no specific funding for this work.*<br><br>**Funded studies**<br>Enter a statement with the following details:<br>• Initials of the authors who received each award<br>• Grant numbers awarded to each author<br>• The full name of each funder<br>• URL of each funder website<br>• Did the sponsors or funders play any role in the study design, data collection and analysis, decision to publish, or preparation of the manuscript?<br>• **NO** - Include this sentence at the end of your statement: *The funders had no role in study design, data collection and analysis, decision to publish, or preparation of the manuscript.*<br>• **YES** - Specify the role(s) played.<br><br>* typeset	
**Competing Interests**<br><br>Use the instructions below to enter a competing interest statement for this submission. On behalf of all authors, disclose any competing interests that could be perceived to bias this work—acknowledging all financial support and any other relevant financial or non-financial competing interests.<br><br>This statement is required for submission and **will appear in the published article** if the submission is accepted. Please make sure it is accurate and that any funding sources listed in your Funding Information later in the submission form are also declared in your Financial Disclosure statement.<br><br>View published research articles from *PLOS ONE* for specific examples.	The authors have declared that no competing interests exist.

**NO authors have competing interests**<br><br>Enter: *The authors have declared that no competing interests exist.*<br><br>**Authors with competing interests**<br><br>Enter competing interest details beginning with this statement:<br><br>*I have read the journal's policy and the authors of this manuscript have the following competing interests: [insert competing interests here]*<br><br>* typeset	
**Ethics Statement**<br><br>Enter an ethics statement for this submission. This statement is required if the study involved:<br><br>• Human participants<br>• Human specimens or tissue<br>• Vertebrate animals or cephalopods<br>• Vertebrate embryos or tissues<br>• Field research<br><br>Write "N/A" if the submission does not require an ethics statement.<br><br>General guidance is provided below. Consult the submission guidelines for detailed instructions. **Make sure that all information entered here is included in the Methods section of the manuscript.**	N/A

Powered by Editorial Manager® and ProduXion Manager® from Aries Systems Corporation

**Format for specific study types**<br><br>**Human Subject Research (involving human participants and/or tissue)**<br>• Give the name of the institutional review board or ethics committee that approved the study<br>• Include the approval number and/or a statement indicating approval of this research<br>• Indicate the form of consent obtained (written/oral) or the reason that consent was not obtained (e.g. the data were analyzed anonymously)<br><br>**Animal Research (involving vertebrate animals, embryos or tissues)**<br>• Provide the name of the Institutional Animal Care and Use Committee (IACUC) or other relevant ethics board that reviewed the study protocol, and indicate whether they approved this research or granted a formal waiver of ethical approval<br>• Include an approval number if one was obtained<br>• If the study involved *non-human primates*, add *additional details* about animal welfare and steps taken to ameliorate suffering<br>• If anesthesia, euthanasia, or any kind of animal sacrifice is part of the study, include briefly which substances and/or methods were applied<br><br>**Field Research**<br><br>Include the following details if this study involves the collection of plant, animal, or other materials from a natural setting:<br>• Field permit number<br>• Name of the institution or relevant body that granted permission	
**Data Availability**<br><br>Authors are required to make all data underlying the findings described fully available, without restriction, and from the time of publication. PLOS allows rare exceptions to address legal and ethical concerns. See the PLOS Data Policy and FAQ for detailed information.	No - some restrictions will apply

Powered by Editorial Manager® and ProduXion Manager® from Aries Systems Corporation

A Data Availability Statement describing where the data can be found is required at submission. Your answers to this question constitute the Data Availability Statement and **will be published in the article**, if accepted.<br><br>**Important:** Stating 'data available on request from the author' is not sufficient. If your data are only available upon request, select 'No' for the first question and explain your exceptional situation in the text box.<br><br>Do the authors confirm that all data underlying the findings described in their manuscript are fully available without restriction?	
**Describe where the data may be found in full sentences. If you are copying our sample text, replace any instances of XXX with the appropriate details.**<br><br>• If the data are **held or will be held in a public repository**, include URLs, accession numbers or DOIs. If this information will only be available after acceptance, indicate this by ticking the box below. For example: *All XXX files are available from the XXX database (accession number(s) XXX, XXX.*).<br>• If the data are all contained **within the manuscript and/or Supporting Information files**, enter the following: *All relevant data are within the manuscript and its Supporting Information files.*<br>• If neither of these applies but you are able to provide **details of access elsewhere**, with or without limitations, please do so. For example:<br><br>*Data cannot be shared publicly because of [XXX]. Data are available from the XXX Institutional Data Access / Ethics Committee (contact via XXX) for researchers who meet the criteria for access to confidential data.*<br><br>*The data underlying the results presented in the study are available from (include the name of the third party*	All relevant data are within the manuscript and its Supporting Information files.

Powered by Editorial Manager® and ProduXion Manager® from Aries Systems Corporation

and contact information or URL).<br>• This text is appropriate if the data are owned by a third party and authors do not have permission to share the data.<br><br>* typeset	
Additional data availability information:	Tick here if the URLs/accession numbers/DOIs will be available only after acceptance of the manuscript for publication so that we can ensure their inclusion before publication.

Powered by Editorial Manager® and ProduXion Manager® from Aries Systems Corporation

Manuscript Click here to access/download;Manuscript;2023-9--4 clean copy.doc

Effects of biofertilizers on the growth, leaf physiological Indices and chlorophyll fluorescence response of spinach seedlings

Beibei Zhang[1,2*], Hui Zhang[1], Di Lu[3], Liping Cheng[3], Jiajia Li[3]

[1] Shaanxi Key Laboratory of Disaster Monitoring and Mechanism Simulating, College of Geography and Environment, Baoji University of Arts and Sciences, Baoji,Shaanxi, China

[2] School of Surveying and Land Information Engineering, Henan Polytechnic University, Jiaozuo, Henan, China

[3] College of Tourism and Management, Pingdingshan University, Pingdingshan, Henan, China

***Corresponding author:**

E-mail: zbb83101@126.com (BZ)

Current Address: Shaanxi Key Laboratory of Disaster Monitoring and Mechanism Simulating, College of Geography and Environment, Baoji University of Arts and Sciences, Baoji, China

Abstract

Chemcial fertilizer as the main strategy for improving the vegetable yields was excessively applied in recent years which led to progressively serious soil problems such as the soil acidification. According the situation, five different biofertilizer treatments [no fertilizer (CK), inoculations of *Bacillus subtilis* (*Bs*, T1), combination of *Bs* and *Bacillus mucilaginosus* (*Bs*+*Bm*, T2), *Bs* and *Bacillus amyloliquefaciens* (*Bs*+*Ba*, T3), and *Bm*+*Ba* (T4)] were conducted to investigate the effect of the growth, leaf physiological indices, and chlorophyll fluorescence of spinach seedlings in the growth chamber. The growth and physiological indices of the spinach seedlings attained a maximum under the T2 treatments. Under the T2 treatment, the ABS/RC (Absorption flux per RC), TR_0/RC (Trapping flux per RC), and ET_0/RC (Electron transport flux per RC) was significantly increased, while the DI_0/RC [Dissipated energy flux per RC (at t=0)] was decreased. The OJIP curve was improved under of the inoculations of fertilizers, and the increasing range was the largest under the T2 treatment. The leaf light response curve (LC) was also significantly increased under the T2 treatment. The plant growth characteristics [leaf length (LL), leaf weight (LW), plant height (PH)] were positively correlated with the J-I-P test chlorophyll fluorescence parameters [PI_{ABS} (Performance index for energy conservation from exciton to the reduction of intersystem electron acceptors), φP_0 (Maximum quantum yield of primary photochemistry), φE_0 (Quantum yield of electron transport), ψ_0 (The probability that a trapped exciton moved an electron in electron transport chain further than QA^-), TR_0/RC, and ET_0/RC] while negatively correlated with φD_0 (Quantum

yield of energy dissipation) and DI_0/RC. The leaf physiological characteristics [SP (soluble protein concentrations), SC (soluble carbohydrate concentrations), Chl a (chlorophyll a), Chl b (chlorophyll b), Chl a+b, Chl a/b, and WP (water potential)] were positively correlated with the J-I-P test chlorophyll fluorescence parameters (PI_{ABS}, φP_0, φE_0, ψ_0, ABS/RC, TR_0/RC, and ET_0/RC) while negatively correlated with φD_0 and DI_0/RC. These results indicated that the combination of *Bs*+*Bm* inoculations promoted the growth of the spinach and improved the adaptability of the vegetable to acid soil while *Ba* inoculation didn’t have any effects to plants.

Keywords: *Bacillus*; Growth; Physiological characters; Chlorophyll fluorescence

Introduction

Spinach (*Spinacia oleracea L.*) is a big-leaf variety, which belonged to the spinach genus Chenopodiaceae (annual herbaceous plant), as an important leafy green vegetable its leaves and shoots that contain large quantities of bioactive compounds, minerals, and vitamins like A, B, and C and nutrients [1,2]. It can scavenge free radicals and has several medical and food applications [3]. The plant has also several antibacterial compounds and folic acid which is useful for the treatment of anemia [4]. In 2020, the global spinach planting area is about 929, 000 hectares and the production is about 31.43 million tons [5,6]. Among them, the global spinach production mainly came from China and Chinese spinach production was accounting for 91.51% of the global output [7]. So, the soil condition seriously affects the growth and yield of spinach. Recent years, fertilization as the main strategy to improve the vegetables' yield, the excessive application of chemical fertilizers has led to progressively serious soil problems such as the soil acidification, while the spinach is sensitive to it [8]. Therefore, biofertilizers have gradually replaced chemical fertilizers to improve soil quality to increase vegetable yield. Biofertilizers are also referred to as bacterial fertilizer, biological fertilizer, and microbial inoculant [9], which contains beneficial microorganisms that provide fertilization for crops through their activities.

SPECIES?

The metabolic activities of microorganisms can improve soil fertility and crop quality, and also increase soil microorganism populations, reduce plant diseases, and enhance plant root activities [10,11]. Among them, *Bacillus* is a type of growth-promoting bacteria that can produce phytase at the rhizosphere of plants. It

has strong resistance to ultraviolet light, high salt, high acid, high heat, and radiation, with the capacity to inhibit bacteria, prevent disease, and increase production [12]. *Bacillus subtilis (Bs)* can improve the stress resistance of plants [13] and the availability of nitrogen and phosphorus in soil [14]. *Bacillus mucilaginosus (Bm)* can convert the unusable phosphorus in soil to available phosphorus for plants. Simultaneously, *Bm* can secrete substances that promote plant growth and development, such as growth hormones, gibberellin, and more [15,16]. *Bacillus amyloliquefaciens (Ba)* has a certain antagonistic effect on bacterial diseases [17].

Photosynthesis is a process through which plants convert captured light energy into biochemical energy [18]. Photosynthetic performance was found to be a very informative indicator, because of its extreme sensitivity to environment [19].Chlorophyll fluorescence measurements as one of photosynthetic performances have become a widely used method to study the functioning of the photosynthetic apparatus and are a powerful tool to study the plant’s response to different environments [20]. Through the analyses of chlorophyll fluorescence parameters, a further elucidation of the light energy absorption, utilization, transfer, and dissipation of plant chloroplast Photosystem I (PSI) and Photosystem II (PSII) processes can be obtained [21]. The chlorophyll fluorescence technique has also been widely investigated for the detection of physiological changes in plants that are caused by bacterial and fungal infections [22]. Chu et al. [23] and Bing et al. [24] revealed that there was significant correlation between chlorophyll fluorescence parameters and the degree of Verticillium wilt infection in cotton leaves. Tung et al. [25] quantitatively

analyzed the degree of infected tobacco (infected, unmanifested infection, healthy) according to Fv/Fm images, which indicated that chlorophyll fluorescence imaging technologies could detect the degree of infected tobacco.

The purpose of this research was to evaluate the variables related to growth, physiological characters and chlorophyll florescence of spinach seedlings in response to different biofertilizers under acid soil condition. The results of this study would be used to give guidance on growth, yield and soil improvement for spinach cultivation in a controlled environment.

Materials and Methods

Experimental materials

The spinach variety *Spinacia oleracea L.*was used as the experimental material. The *Bacillus subtilis* (*Bs*), *Bacillus mucilaginosus* (*Bm*), and *Bacillus amyloliquefaciens* (*Ba*) selected the original strains produced by Lvlong Biotecnology Co., Ltd., which contained >200 million live bacteria per gram. The fertilizer components were the *Bacillus* and its metabolites, and the rate of miscellaneous bacteria <3%.

No specific permissions were required for research locations and the studies did not involve endangered or protected species. All the authors consent for publication.

Growth conditions and experiment design

The soil came from the Qinxi demonstration garden in Taibai County whose nutrients were comprised of 33.35 $g \cdot kg^{-1}$ organic matter, 2.79 $g \cdot kg^{-1}$ total nitrogen, 1.51 $g \cdot kg^{-1}$ total phosphorus, and 10.75 $mg \cdot kg^{-1}$ available phosphorus, and a pH of 5.2.

The experiments were carried out in some growth chambers (FytoScope FS 130-WIR, Brno, Czech) under a 12 h day/night photoperiod at temperatures of 25/15°C, respectively, a photosynthetically active radiation (PAR) of 300 μmol/(m^2·s), and humidity of 60%. According to the experiment design, the growth chambers (FytoScope FS 130-WIR) could monitor, control and adjust the temperature, PAR and humidity automatically. Five different biofertilizer treatments [no fertilizer (CK), inoculations of *Bacillus subtilis* (*Bs*, T1), combination of *Bs* and *Bacillus mucilaginosus* (*Bs*+*Bm*, T2), *Bs* and *Bacillus amyloliquefaciens* (*Bs*+*Ba*, T3), and *Bm*+*Ba* (T4)] were conducted. The experimental design was a factorial scheme with 4 replicates per treatment.

when was applied?

The spinach seeds were soaked in deionized water for 12 h and placed in a 4°C refrigerator for 24 h to promote germination. The germinated seeds were placed on a seedling tray, covered with nutritious soil, and germinated in the dark in a 25°C incubator. Following seven days of treatment, the seedlings were transferred to pot (Ø25 cm x 20 cm high) filled with soil 18 cm deep, and 10 seedlings were planted in each pot. For this experiment, 90 kg·km^{-2} of phosphate fertilizer (potassium dihydrogen phosphate) was used as base fertilizer with biofertilizer 0.5 kg·km^{-2}.

Measurement of growth indices

For this experiment, all of the characteristics of the spinach leaves were measured at 7th day after treatments. The plant height (PH), leaf length (LL) and leaf weight (LW) were measured with a scale.

Measurement of leaf physiological indices

After 7 to 10 days treatment, the leaf soluble protein concentration (SP) was determined by the Coomassie brilliant blue (CBB) method at 595 nm using a DR6000 spectrophotometer [26], whereas the soluble carbohydrate concentration (SC) was determined using an anthrone method at 620 nm [27]. The leaf Chlorophyll a (Chl a) and Chlorophyll b (Chl b) concentrations were determined by Litchtenthaler [28] at 645 and 663 nm, whereas the leaf malondialdehyde (MDA) was determined by the thiobarbituric acid method [29]. The leaf water potential (WP) (ψ) was measured by dew point potentiometer (WP4, Decagon Devices, Pullman, USA).

Chlorophyll fluorescence measurements

Prior to the fluorometer measurements, the leaves were dark-adapted for 20 min and then measured using a FluorPen FP 100Max hand fluorescence meter (Photon Systems Instruments, Brno, Czech), and calculated according to the JIP-test algorithm proposed by Strasser et al. [30] (Table 1).

To compare the OJIP curve with the normalized Chlorophyll fluorescence (CF) transient curve between OJ and OK, the following formula (1), (2), (3), (4), (5) and (6) were used.

$$V_t=(F_t-F_0)/(F_m-F_0) \quad (1)$$

$$\Delta V_t=V_t^{TR}-V_t^{CK} \quad (2)$$

$$W_{OJ}=(F_t-F_0)/(F_j-F_0) \quad (3)$$

$$\Delta W_{OJ}=W_{OJ}^{TR}-W_{OJ}^{CK} \quad (4)$$

$$W_{OK}=(F_t-F_0)/(F_K-F_0) \quad (5)$$

$$\Delta W_{OK}=W_{OK}^{TR}-W_{OK}^{CK} \quad (6)$$

Table 1 Parameters of chlorophyll fluorescence

Parameters and Formula	Meaning
F_0	minimum fluorescence
F_m	maximum fluorescence
F_J	F_t at Time 2 ms
F_I	F_t at Time 30 ms
F_t	The relative fluorescence intensity at Time t
$F_v=F_m-F_0$	variable fluorescence
$nF_v=F_v/F_0$	variable to initial fluorescence ratio
$V_I=(F_J-F_0)/(F_m-F_0)$	Relative variable fluorescence at the I-step
$V_J=(F_J-F_0)/(F_m-F_0)$	Relative variable fluorescence at the J-step
M_0	Approximated initial slope (in ms^{-1}) of the fluorescence transient V= f(t)
PI_{ABS}	Performance index (potential) for energy conservation from exciton to the reduction of intersystem electron acceptors
ABS/RC	Absorption flux (of antenna Chls) per RC
$TR_0/RC=M_0(1/V_J)$	Trapping flux (leading to QA reduction) per RC
$ET_0/RC=M_0(1/V_J)\Psi o$	Electron transport flux (further than QA^-) per RC
$DI_0/RC=(ABS/RC-TR_0/RC)$	Dissipated energy flux per RC (at t = 0)
$\varphi P_0=1-F_0/F_m$	Maximum quantum yield of primary photochemistry (at t = 0)
$\varphi E_0=(1-F_0/F_m)(1-V_J)$	Quantum yield of electron transport (at t = 0)
$\Psi_0= ET_0/Tr_0=1-V_J$	The probability that a trapped exciton moved an electron in electron transport chain further than QA^-（t=0）
$\varphi D_0=F_0/F_m$	Quantum yield (at t = 0) of energy dissipation

The leaf light-response curve (LC) measurement, based on pulse modulated fluorometry (PAM) was designed using seven photosynthetic photon flux densities (PPFD) (10，20，50，100，300，500, and 1000 μmol/(m^2·s)) to acquire chlorophyll fluorescence parameter changing curves (Ft, QY) relating the rate of photosynthesis.

Statistical analysis of data

All collected data were subjected to one-way ANOVA analysis using SPSS (SPSS software version 22.0, Chicago, Illinois, USA). Differences between means

were compared by Bonferroni test at P<0.05. The correlations between parameters were determined using Pearson's simple correlation test function in SPSS.

Results

Effects of biofertilizers on growth and physiological indices of spinach seedlings

There were significant differences in plant height (PH), leaf length (LL), leaf

which?

weight (LW), and other growth parameters of the spinach seedlings under the five different treatments (F=54.37, 13.30, and 46.03, respectively; P<0.01), where the data

this sentence it is not true because T2 is similar to T1

of growth indices was maximum under the T2 treatment (Table 2). There were significant differences among the treatments in the leaf physiological indices (Table

???? this sentence it is not true for all physiological parameters

3). And the T2 treatment had the greatest impact on the leaf physiological responses.

this sentence has not sense!

Compared with the F value, the difference in soluble sugar (SC) was the highest (F=118.35; P<0.01).

Table 2 Differences of the growth characteristics under 5 different biofertilizer treatments of spinach

Treatments	Growth characters		
	Plant height (PH, cm)	Leaf length (LL, cm)	Leaf weight (LW, g/10 plants)
None, CK	3.53±0.15 b	2.62±0.26 b	0.95±0.01 c
Bs, T1	5.23±0.09 a	4.08±0.05 a	2.08±0.02 a
Bs+*Bm*, T2	5.27±0.09 a	4.15±0.09 a	2.69±0.02 a
Bm+*Ba*, T3	5.05±0.03 a	3.73±0.18 a	1.47±0.02 b
Bs+*Ba*, T4	5.07±0.03 a	3.89±0.19 a	1.74±0.01 b
F value	54.37**	13.30**	46.03**

Values presented in each column of table have a mean ±standard deviation. The last portion of table refers to the F value. * P<0.05, ** P<0.01.

Table 3 Differences of leaf physiological indexes under 5 different biofertilizer treatments of spinach

as it is possible that 0.58 is ab and 0.60 is b????

Treatments	Leaf physiological indexes SP (mg/g FW)	SC (mg/g FW)	MDA (mg/gFW)	Chl a (mg/g FW)	Chl b (mg/g FW)	Chl a+b (mg/g FW)	Chl a/b (mg/g FW)	WP(Ψ, Mpa)
None, CK	1.83±0.26c	0.73±0.05d	5.49±0.51a	1.16±0.03e	0.36±0.03c	1.52±0.03e	3.25±0.30b	-6.57±0.25d
Bs, T1	3.31±0.20b	2.26±0.06b	1.80±0.13b	2.73±0.22b	0.58±0.06ab	3.30±0.15b	4.91±0.83a	-4.23±0.12b
Bs+*Bm*, T2	4.14±0.04a	3.59±0.13a	1.25±0.14b	3.23±0.04a	0.60±0.03b	3.83±0.07a	5.36±0.25a	-2.79±0.22a
Bm+*Ba*, T3	2.37±0.08c	1.59±0.11b	4.82±0.31a	1.70±0.07d	0.40±0.01c	2.11±0.07d	4.21±0.09ab	-5.53±0.26c
Bs+*Ba*, T4	3.28±0.22b	2.23±0.11c	2.09±0.15b	2.29±0.11c	0.47±0.02bc	2.75±0.09c	4.91±0.43a	-4.79±0.12b
F value	24.48**	118.35**	44.95**	50.63**	8.80**	118.34**	203.88**	48.72**

Values presented in each column of table have a mean ±standard deviation. The last portion of table refers to the F value. * P<0.05, ** P<0.01. SP: soluble protein concentrations, SC: soluble carbohydrate concentrations, MDA: malonaldehyde concentrations, Chl: chlorophyll, WP: water potential, FW: fresh weight.

Effect of biofertilizers on chlorophyll fluorescence parameters of spinach seedlings

According to the values of the basic fluorescence parameters (Table 4), the F_0

this sentence it is not true

value of the T2 treatment was the highest, which was 41.29% higher than that of CK. Further, the F_m value of the T2 treatment was 17751 higher than that of CK, and there was a significant difference in F_V value under the five treatments. The relative variable fluorescence value (V_I) at 30 ms was highest under the T2 treatment. In terms

it is not true

this sentence has not sense

of the F_v/F_0 value, the T2 treatment remained the largest. Compared with the F value, the difference of F_0 was the largest (F=112.57; P < 0.01).

Table 4 Differences of leaf chlorophyll fluorescence intensity under 5 different biofertilizer treatments of spinach

Treatments	Leaf chlrophyll fluorescence intensity (relative unit)					
	F_0	F_m	F_v	V_J	V_I	$F_v/F_0(nF_v)$
None, CK	7087±188b	28967±1440c	21880±1628c	0.46±0.06a	0.76±0.01bc	4.07±0.03b
Bs, T1	9233±568ab	43142±1342ab	33909±1286ab	0.54±0.03ab	0.82±0.01ab	3.67±0.11a
Bs+*Bm*, T2	10013±10ab	46718±1126a	36705±1136a	0.47±0.00ab	0.85±0.02a	4.08±0.11a
Bm+*Ba*, T3	9071±108ab	31861±131bc	22790±23bc	0.47±0.01b	0.76±0.00c	2.51±0.41b
Bs+*Ba*, T4	9786±38a	41549±1544ab	31763±1506abc	0.46±0.00ab	0.77±0.01bc	3.25±0.24a
F value	112.57**	31.13**	24.01**	2.48	3.85*	8.3**

The values presented in each column of table have a mean ±standard deviation. The last part of table refers to the F value. * P<0.05, ** P<0.01.

Table 5 Differences of J-I-P test parameters under 5 different biofertilizer treatments of spinach leaf

Treatments	J-I-P test parameters (relative unit)					
	M_0	PI_{ABS}	ABS/RC	TR_0/RC	ET_0/RC	DI_0/RC
None, CK	0.91±0.03ab	0.38±0.29c	2.20±0.08ab	1.37±0.06ab	0.46±0.04b	0.82±0.06a
Bs, T1	1.08±0.07a	1.24±0.21ab	2.57±0.03a	2.02±0.01ab	0.94±0.03a	0.55±0.02c
Bs+*Bm*, T2	1.00±0.02a	1.70±0.23a	2.59±0.03a	2.08±0.04a	1.07±0.01a	0.51±0.01b
Bm+*Ba*, T3	0.67±0.01ab	1.41±0.01bc	2.01±0.00b	1.43±0.02b	0.76±0.01ab	0.57±0.01ab
Bs+*Ba*, T4	0.83±001b	1.61±0.03bc	2.35±0.08ab	1.80±0.01ab	0.96±0.01ab	0.55±0.00bc
F value	7.66**	4.09*	2.88	3.97*	2.04	8.16**

The values presented in each column of table have a mean ±standard deviation. The last part of table refers to the F value. * P<0.05, ** P<0.01.

There were significant differences between the M_0 and PI_{ABS} values under the five different treatments (F=7.66 and 4.09, respectively; P<0.05) (Table 5). The T2

this is not true

treatment had the greatest effect on the specific activity of each reaction center (RC), and there was a significant difference between the TR_0/RC and DI_0/RC. However,

there was no significant difference in the ABS/RC and ET_0/RC between the treatments. The quantum yield (φP_0), efficiency (φE_0), and ψ_0 values were highest under the T2 treatment, and with the exception of ψ_0, there were significant differences (Table 6) ($P<0.01$). On the contrary, the value of φD_0 was lowest under the T2 treatment.

the sentence is not true

Table 6 Differences of leaf chlorophyll fluorescence yield and efficiency under 5 different biofertilizer treatments of spinach

Treatments	Yield and efficiency			
	φP_0(F_v/F_m)	φE_0	Ψ_0	φD_0
None, CK	0.62±0.01c	0.22±0.01ab	0.34±0.01ab	0.37±0.01ab
Bs, T1	0.78±0.01ab	0.37±0.01a	0.47±0.01a	0.21±0.00c
Bs+Bm, T2	0.80±0.00a	0.41±0.06b	0.52±0.05a	0.19±0.00bc
Bm+Ba, T3	0.72±0.0bc	0.38±0.03ab	0.53±0.02b	0.29±0.03a
Bs+Ba, T4	0.76±0.01ab	0.41±0.00ab	0.54±0.00ab	0.24±0.01bc
F value	6.03**	2.48	2.71	6.02**

The values presented in each column of table have a mean ±standard deviation. The last part of table refers to the F value. * $P<0.05$, ** $P<0.01$.

Effect of biofertilizers on transient analysis of prompt fluorescence OJIP of spinach seedlings

The transient curve of OJIP was shown in Fig 1 and the OJIP curves of the five treatments were similar to those reported by Strasser et al. [31]. In Fig 1(A), compared with CK, the fluorescence trend of other treatments gradually increased. Among them, the fluorescence value of the T2 treatment was the largest, which signified that it had a greater effect on the photosynthetic chemical rate of the leaves. As shown in Fig 1(B), the difference of relative variable fluorescence (Vt) at point J (2 ms) was the

it is not true

this is not evident in Figure 1B

largest. This was better illustrated in Fig 1(C) ΔV_t. Over time the difference between the treatments increased initially, then decreased, and the difference of the initial slope during the O-J stage curve was maximized. It can be seen in Figs 1 D and E, while there was an obvious positive K band under the T1 and T2 treatments, and a negative growth trend under the T3 and T4 treatments. The transient double normalization of OJIP between F_0 and F_K and the difference of double normalization between the treatments and the control group were regarded as the L band. As shown in Figs 1 F and G, the W_{OK} value of the T2 treatment was higher, and there was a significant higher positive L band.

Effect of biofertilizers on the difference of the Light response Curve (LC) of the spinach seedlings

In Fig 2, compared with the five treatments, the quantum yield (QY) of leaves under the T2 treatment attained a maximum at about 20 μmol/(m^2·s) PPFD, which indicated that the light compensation point (LCP) was around 20 μmol/(m^2·s) PPFD. The maximum value of the other treatments was before 10 μmol/(m^2·s) PPFD, indicating that the LCP was earlier than 10 μmol/(m^2·s) PPFD.

Correlations between growth, leaf physiological indices and chlorophyll fluorescence

Through correlation analyses, the indices of growth, physiology, and chlorophyll fluorescence of leaves were quantified. Plant growth indices (LL, LW, PH) were positively correlated with chlorophyll fluorescence parameters (PI_{ABS}, φP_0, φE_0, ψ_0, TR_0/RC, ET_0/RC), while which was negatively correlated with φD_0 and DI_0/RC. The

physiological indices of the leaves (SP, SC, Chl a, Chl b, Chl a+b, Chl a/b, and WP) were positively correlated with PIABS, φP_0, φE_0, ψ_0, ABS/RC, TR_0/RC and ET_0/RC, and negatively correlated with φD_0 and DI_0/RC. The leaf MDA was significantly positively correlated with φD_0 and DI_0/RC, and positively correlated with other chlorophyll fluorescence parameters (Table 7).

Table 7 Correlations between the leaf physiological characteristics and chlorophyll fluorescence parameters

Index	LW	LL	PH	SP	SC	Chl a	Chl b	Chl a+b	Chl a/b	MDA	WP
PI_{ABS}	0.75**	0.84**	0.92**	0.76**	0.81**	0.73**	0.55*	0.73**	0.68**	-0.71**	0.75**
φP_0	0.90**	0.89**	0.91**	0.89**	0.90**	0.93**	0.80**	0.93**	0.74**	-0.91**	0.90**
φE_0	0.58*	0.78**	0.89**	0.60*	0.65**	0.56*	0.38	0.55*	0.58*	-0.55*	0.58*
Ψ_0	0.74**	0.86**	0.95**	0.75**	0.79**	0.74**	0.56*	0.74**	0.68**	-0.72**	0.74**
φD_0	-0.90**	-0.89**	-0.91**	-0.89**	-0.90**	-0.93**	-0.80**	-0.93**	-0.74**	0.91**	-0.90**
ABS/RC	0.77**	0.51	0.40	0.78**	0.73**	0.83**	0.80**	0.84**	0.54*	-0.85**	0.78**
TR_0/RC	0.90**	0.74**	0.69**	0.90**	0.88**	0.94**	0.86**	0.96**	0.68**	-0.95**	0.90**
ET_0/RC	0.89**	0.88**	0.90**	0.91**	0.92**	0.91**	0.76**	0.92**	0.75**	-0.91**	0.90**
DI_0/RC	-0.80**	-0.90**	-0.97**	-0.78**	-0.82**	-0.80**	-0.65**	-0.81**	-0.70**	0.76**	-0.79**

* $P<0.05$** $P<0.01$. LL: Leaf length, LW: Leaf weight, PH: Plant height, SP: Soluble protein concentrations, SC: Soluble carbohydrate concentrations, MDA: malonaldehyde concentrations, Chl: chlorophyll, WP: Water potential.

Discussion

Physiological indices of growth and leaves

In terms of plant phenology and biological characteristics, plant growth, leaf chlorophyll concentration, and other physiological indices were critical influencing

factors [32]. The results of Yan et al. [33] revealed that the application of biofertilizer could effectively improve the plant height of pakchoi. In this study, the inoculations of biofertilizer effectively improved the plant height, leaf length, and leaf weight of

this is not true

spinach. The growth status of plants with the inoculations of *Bs* or *Bm* was more dominant and it was similar to the results of Samia et al. [34].

The leaf soluble protein (SP), soluble carbohydrate (SC), and water potential (WP) were closely related to plant metabolism. Chlorophyll (Chl) was an important substance in plant photosynthesis, and malondialdehyde (MDA) can reflect the degree of membrane lipid peroxidation [35]. In this research, When *Bs* and *Bm* (T2) were inoculated together, the leaf physiological indices of spinach seedling (SP, SC, Chl a,

this is not treu for Chl b, and Chla/b

Chl b, Chl a+b, Chl a/b, and WP) were significantly enhanced. Wang et al. [36] found that the application of biofertilizer could effectively increase the chlorophyll concentration of winter wheat at the different growth stages. The application of

what means this sentence?

phosphate fertilizer can promote the synthesis of chlorophyll in leaves [37].

For this study, the leaf chlorophyll concentration treated with the combination of *Bs*+*Bm* treatment (T2) was the highest, whereas when *Bs* or *Bm* were paired with *Ba* there was less chlorophyll. It might be possibly explained that it could convert ineffective nitrogen or phosphorus into available nitrogen or phosphorus in the soil and improved the increase in N fixation and P solubilization of the plants after the application of *Bs* or *Bm*. Thus promoting the synthesis of ATP (Adenosine

this sentence has not sense

triphosphate) and NADPH (nicotinamide adenine dinucleotide phosphate) in leaves then promoting the growth. Likewise, after inoculation it also could affect the

regulation of phytohormone biosynthesis pathways; modulate ethylene levels in plants

it is not possible to hypotesize because authors did not determine these moelcules

and the launch of host plants' systemic tolerance [38]. The inoculation of *Ba* didn't have more effects. The reason for the analysis may be that *Ba* mainly targets the repair effect of plant facing to bacterial and fungal infections, and the plants used in the experiment were not infected, so the promoting effect on plant growth is not significant. When plants were under stress, the MDA concentration was typically an important indicator of membrane lipid peroxidation, which reflected the harmful effects of stress on plant cells and tissues [39]. In this study, the concentration of MDA in leaves treated with biofertilizer was decreased which mean the plant was in a

but the problem is: why MDA aoumnt is so high in controls?

suitable environment.

Leaf chlorophyll fluorescence

In this research, the values of F_0 and Fm were enhanced, which indicated that the *Bs* and *Bm* promoted an increase in the size of leaf PSII antenna and a decrease in the non-radiative dissipation of chlorophyll in PSII antenna, thus increasing the capacity of leaves to capture light energy [40]. We observed higher Fv and F_v/F_0 (nF_v) values under the combination of *Bs*+*Bm* treatment, which signified an increase in the efficiency of supplying electrons to PSII reaction centre (RCS) and the photosynthetic quantum conversion rate of PSII RCS. This translated to less energy being used for non-photochemical dissipation in the dark adaptation process [41].

In this experiment, the ABS/RC values of the leaves increased, indicating that after inculcating *Bs*+*Bm* the size of active RCS was increased, which led to a higher number of active PSII reaction centers and enhanced dark accumulation [20]. Under

the action of the *Bs*+*Bm*, the TR_0/RC value increased, which reflected the higher electron capture rate of RC, where more QA (the primary electron acceptor in Photosystem II) was converted to QA^-. This resulted in an increase in the electron transfer energy (ET_0/RC), thus reducing the energy dissipated by non-photochemical activities (DI_0/RC) [42,43].

The φP_0 (Fv/Fm) represented the maximum quantum yield of PS Ⅱ, and the ψ_0 value reflected the electron transfer efficiency, from QA^- to QB, whereas φE_0 reflected the quantum yield of electron transport. The increase of φP_0, ψ_0, and φE_0 indicated that the combination of *Bs*+*Bm* promoted the redox reaction following QA, which resulted in an increase of the electron transfer rate between QA^- and QB [44].

Oukarroum et al. [39] regarded the double normalization of the OJIP transient between the peak value of 0.05 to 2 ms, and the difference of double normalization between the treatment and the control group as the K-band. The change of the chlorophyll fluorescence curve was closely related to the physiological morphology of plants [45]. Changes in the O-J segment were related to an increased number of inactive reaction centers, or the energy transfer from the LHC II (Light-harvesting Complex) to PS II RCS [46]. The K and L bands reflected the connection between the S state of the PSII oxygen evolution complex (OEC) and PS II unit, as well as the energy connection between the PS II units [47,48].

An increase in the J-I segment could reflect a decrease in the relative number of active PQ (plastoquinone) molecules that was reduced by each active RCS of PSII [48]. Further, changes in the I-P segment were closely related to the pool of electron

receptors (ferredoxin and NADPH) at the end of the PSI, signifying the kinetic flow rate to the electron receptors at the end of the PSI [49]. In this experiment, the OJIP transient curves of the spinach seedlings were affected by biofertilizer (Fig.1).

Under the T2 treatment, the relative fluorescence intensity of chlorophyll in the O-J segment exhibited a larger value, indicating that the population of active PSII centers decreased, whereas the QA^- accumulated massively. Fig.1 (E and G) showed that there were obvious K bands and positive L bands under the T2 treatment, which indicated that the PSII had an inhibitory effect on the OEC. This resulted in a weakening of the connectivity between PSII and OEC and a decreased energy connection between the PSII units. This might due to the variable light and ventilation that was present at different locations in the incubator, which might have led to PSI receptor side damage and chlorophyll protein denaturation in some plant leaves [50]. In addition, the fluorescence of J-I and I-P segments remained large under the T2 treatment, which indicated that the relative number of PQ decreased, while there was an increase in the pool of electron receptors (ferredoxin and NADPH) at the end of PSI, which led to a higher kinetic flow rate to the electron receptors at the end of PSI. Combined with PI_{ABS}, the higher PI_{ABS} values under combination of *Bs* and *Bm* treatment indicated increases in the density of active PSII centers, the efficiency of photoreactions, and efficiency of biochemical dark redox reactions, as well as the production and utilization of NADPH [51].

Conclusions

In this study, the role of different biofertilizers was analyzed in spinach seedling. It was observed that the inoculations of biofertilizers *Bs* or *Bm* stabilized the plant cell membrane and increased the rate of photosynthesis which led toward the improvement of plant growth. Acid soil reduced the growth of plant and increased the amount of ROS production which disrupted the plant metabolism. The application of biofertilizers triggered osmotic adjustment, maintained the production of antioxidants, improved the leaf chlorophyll fluorescence and promoted the growth in plants. The T2 treatment significantly ($p<0.05$) increased the growth, physiological index and leaf chlorophyll fluorescence of spinach. It could be concluded that combined inoculations of *Bs*+*Bm* was a better strategy for reducing the negative effects of acid soil on spinach. Due to limited experimental conditions, the experiment was only analyzed at 300 μmol/(m^2•s) light conditions without designing the multiple light conditions. In the next experiment, the impact of biofertilizer inoculation on spinach under multiple light conditions will be explored. Likewise, future research also should endeavor to analyze the utilization of mineral elements in the soil, prevent soil acidification caused by the excessive accumulation of mineral elements and spinach quality to control the fertilization concentrations of microorganisms, so as to provide an improved theoretical basis for spinach production and obtained the great economic benefits.

Declarations

- **Ethics approval and consent to participate**

The research passed the ethics approval and it was consent to participate.

- Consent to publish

All the authors consent for publication.

- Availability of data and materials

The data and materials are available.

- Competing interests

All the authors declare no competing interests.

- Authors' Contributions

D. L. and J. J. L. prepared the materials. B. B. Z designed the study. L. P. C. did the data analysis. B. B. Z and H. Z. wrote the main manuscript text. All authors discussed the results and commented on the paper.

- Acknowledgements

The authors are grateful to Jing Qi, Jiaxuan Wang, Jiayi Han and Xiaoying Hu for assistance with the data measuring.

- Data availability

All data are available upon request by contact with the corresponding author. The minimal data set as a Supporting Information file uploaded.

References

1. Xu CP, Leskovar DI. Effects of A. nodosum seaweed extracts on spinach growth, physiology and nutrition value under drought stress. Sci Hortic 2015; 183: 39-47.
2. Roberts JL, Moreau R. Functional properties of spinach (Spinacia oleracea L.) phytochemicals and bioactives. Food funct 2016; 7(8): 3337-3353.

3. Bunea A, Andjelkovic M, Socaciu C, Bobis O, Neacsu M, Verhé R, Camp JV. Total and individual carotenoids and phenolic acids content in fresh, refrigerated and processed spinach (Spinacia oleracea L.). Food Chem 2008; 108 (2): 649-656.
4. Atti AR, Palmer K, Volpato S, Zuliani G, Winblad B, Fratiglioni L. Anaemia increases the risk of dementia in cognitively intact elderly. Neurobiol Aging 2006; 27 (2): 278-284.
6. Wang M. Analysis of spinach planting area, output and price in the world and China in 2020. Industrial Information Network, 2020-04-08: https://www.chyxx.com/industry/202104/943941.html.
7. Awika HO, Solorzano J, Cholula U, Shi AR, Enciso J, Avila CA. Prediction modeling for yield and water-use efficiency in spinach using remote sensing via an unmanned aerial system. Smart Agr Tech 2021; 1: 100006.
8. Dawson CJ, Hilton J. Fertilizer availability in a resource-limited world: production and recycling of nitrogen and phosphorus. Food Policy 2012; 36: 14-22.
9. Osman M, El-Sheekh MM, El-Naggar AH, Gheda SH. Effect of two species of cyanobacteria as biofertilizers on some metabolic activities, growth, and yield of pea plant. Biol Fert Soils 2010; 46(8): 861-875.
10. Cao EH, Hou XW, Li GY, et al. Effect of combination bacteria on soil physicochemical properties and soil microbial activity by pot tomato experiments. Ecology and Environmental Sciences 2011; 20(5): 875-880.

11. Dinesh R, Srinivasan V, Hamza S, Manjusha A. Short-term incorporation of organic manures and biofertilizers influences biochemical and microbial characteristics of soils under an annual crop [Turmeric (*Curcuma longa L.*)]. Bioresource Techno 2010; 101(12): 4697-4702.

12. Guérout-Fleury AM, Shazand K, Frandsen N. Antibiotic-resistance cassettes for *Bacillus subtilis*. Gene 1995; 167(1-2): 335-336.

13. Pliego C, Ramos C. Screening for candidate bacterial biocontrol agents against soilborne fungal plant pathogens. Plant Soil 2010; 340(1): 505-520.

14. Idriss EE, Makarewicz O, Farouk A, Rosner K, Greiner R, Bochow H, Richter T, Borriss R. Extracellular phytase activity of Bacillus amyloliquefaciens FZB45 contributes to its plant-growth-promoting effect. Microbiology 2002; 148(7): 2097-2109.

15. Liu SN,Tang WZ, Yang F Meng J, Li XZ. Influence of biochar application on potassium-solubilising *Bacillus mucilaginosus* as potential biofertilizer. Prep Biochem Biotech 2016; 1: 32-37.

16. ZhaoY, Zhang XB, GuoW. Physiological and biochemical characteristics and capacities of potassium-releasing of *Bacillus mucilaginosus* screened from different soils. Ecol Environ Sci 2009; 6: 2283-2286.

17. Arrebola E, Jacobs R, Korsten L. Iturin A is the principal inhibitor in the biocontrol activity of Bacillus amyloliquefaciens PPCB004 against postharvest fungal pathogens. J Appl Microbiol 2010; 108(2):386-395.

18. Maxwell K, Johnson GN. Chlorophyll fluorescence e a practical guide. J Exp Bot 2000; 51: 659-668.
19. Massacci A, Nabiev SM, Pietrosanti L, Nematov SK, Chernikova TN, Thor K, Leipner J. Response of the photosynthetic apparatus of cotton (*Gossypium hirsutum*) to the onset of drought stress under field conditions studied by gas-exchange analysis and chlorophyll fluorescence imaging. Plant Physiol Bioch 2008; 46: 189-195.
20. Mehta P, Jajoo A, Mathur S, Bharti S. Chlorophyll a fluorescence study revealing effects of high salt stress on Photosystem II in wheat leaves. Plant Physiol Bioch 2010; 48: 16-20.
21. Li PM, Gao HY, Reto JS. Application of the fast chlorophyll fluorescence induction dynamics analysis in photosynthesis study. J Pla Physiol Mol Biol 2005; 31(6): 559-566.
22. Zaidi A, Ahmad E, Khan MS, Saif S, Rizvi A. Role of plant growth promoting rhizobacteria in sustainable production of vegetables: Current perspective. Sci Hortic 2015; 193: 231-239.
23. Chu Z, Xin W, Li Y, Yu H, Li J, Lu Y, Li H, Bo O. Genomic Organization, Phylogenetic and Expression Analysis of the B-BOX Gene Family in Tomato. Front Plant Sci 2016; 10(7): 1522.
24. Bing C, Wang KR, Li SK, Xiao CH, Wang K. Monitoring Cotton Field with Suspected Verticillium wilt Using Satellite Remote Sensing with TM Satellite Image as an Example. Acta Agron Sinica 2012; 38(1):129-139.

25. Tung J, Goodwin PH, Hsiang T. Chlorophyll fluorescence for quantification of fungal foliar infection and assessment of the effectiveness of an induced systemic resistance activator. Eur J Plant Pathol 2013; 136(2): 301-315.

26. Bradford MM. Arapid and sensitive method for the quantitation of microgram quantities of protein utilizing the principle of proteindye binding. Anal Biochem 1976; 72: 248-254.

27. Jermyn MA. Increasing the sensitivity of the anthrone method for carbohydrate. Anal Biochem 1975; 68(1): 332-335.

28. Litchtenthaler HK, Wellburn AR. Determinations of total carotenoids and chlorophylls a and b of leaf extracts in different solvents. Biochem Soc T 1983; 11(5): 591-592.

29. Draper HH, Squires EJ, Mahmoodi H,Wu J, Agarwal S, Haley M. A Comparative evaluation of thiobarbituric acid methods for the determination of malondialdehyde in biological materials. Free Radical Bio Med 1993; 15(4): 353-363.

30. Strasser RJ, Tsimilli-Michael M, Qiang S, Goltsev V. Simultaneous in vivo recording of prompt and delayed fluorescence and 820-nm reflection changes during drying and after rehydration of the resurrection plant Haberlea rhodopensis. BBA-Biomembranes 2010; 1797: 1313-1326.

31. Strasser RJ, Tsimilli-Michael M, Srivastava A. Analysis of the chlorophyll a fluorescence transient. In: Papageorgiou, G.C., Govindjee (Eds.), Chlorophyll

Fluorescence: a Signature of Photosynthesis. Kluwer Academic Publishers Press, Netherlands 2004; 321-362.

32. Bennie J, Davies TW, CruseD, et al. Ecological effects of artificial light at night on wild plants. J Eco 2016; 104(3): 611-620.

33. Yan DY, He XY, Li YG. Effect of compound microbial fertilizer on the production of brassica chinensis. J Anhui Agri 2017; 45(14): 33-34.

34. Sami AA, Salwa MA. Application of trichoderma Harzianum T22 as a biofertilizer poteneial in maize growth. J Plant Nutr 2014; 37(1): 30-49.

35. Xi ZM, Wang ZZ, Fang YL, Hu ZY, Hu Y, Deng MM, Zhang ZW. Effects of 24- epibrassinolide on antioxidation defense and osmoregulation systems of young grapevines (V. vinifera L.) under chilling stress. Plant Growth Regul 2013; 71: 57-65.

36. Wang JW, Zhao JJ, Li GL. Effects of new compound microbial fertilizer on physiological indexes of winter wheat. J South Agri 2018; 49(10): 1953-1958.

37. Li FM, Song QH, Liu HS, Li FR, Liu XL. Effects of pre-sowing irrigation and phosphorus application on water use and yield of spring wheat under semi-arid conditions. Agr Water Manage 2001; 49(3): 173-183.

38. Awan ZA, Shoaib A. Combating early blight infection by employing Bacillus subtilis in combination with plant fertilizers. Curr Plant Biol 2019; 20: 100125.

39. Han YY, Fan SX, Qiao Z, Wang Y. Effect of heat stress on the MDA, proline and soluble sugar content in leaf lettuce seedlings. Agri Sci 2013; 4(5): 112-115.

40. Oukarroum A, Madidi SDE, Schansker G. Probing the responses of barley cultivars (Hordeum vulgare L.) by chlorophyll a fluorescence OLKJIP under drought stress and re-watering. Environ Exp Bot 2007; 60(3): 438-446.

41. Spoustova P, Synkova H, Valcke R. Chlorophyll a fluorescence as a tool for a study of the Potato virus Y effects on photosynthesis of nontransgenic and transgenic Pssuipt tobacco. Photosynthetica 2013; 51(2): 191-201.

42. Yiotis C, Manetas Y. Sinks for photosynthetic electron flow in green petioles and pedicels of Zantedeschia aethiopica: evidence for innately high photorespiration and cyclic electron flow rates. Planta 2010; 232: 523-531.

43. Tang YP, Sun X, Wen T, Liu MJ, Yang MY, Chen XF. Implications of terminal oxidase function in regulation of salicylic acid on soybean seedling photosynthetic performance under water stress. Plant Physiol Bioch 2017; 112: 19-28.

44. Lebkuecher JG, Haldeman KA, Harris CE, Holz S, Joudah SA, Milton DA. Development of photosystem II activity during irradiance of etiolated Helianthus (Asteraceae) seedlings. Am J Bot 1999; 86(8): 1087-1092.

45. Malaspina P, Giordani P, Pastorino G, Modenesi P, Mariotti MG. Interaction of sea salt and atmospheric pollution alters the OJIP fluorescence transient in the lichen Pseudevernia furfuracea (L.) Zopf. Ecol Indic 2015; 50, 251-257.

46. Tomar RS, JajooA. A quick investigation of the detrimental effectsof environmental pollutant polycyclic aromatic hydrocarbon fluo-ranthene on the

photosynthetic efficiency of wheat (Triticum aestivum). Ecotoxicology 2013; 22: 1313-1318.

47. Dąbrowski P. BaczewskaAH, Pawluśkiewicz B. Prompt chlorophyll a fluorescence as a rapid tool for diagnostic changes in PSII structure inhibited by salt stress in Perennial ryegrass. J Photoch Photobio B 2016; 157: 22-31.

48. Paunov M, Koleva L, Vassilev A. Effects of different metals on photosynthesis: cadmium and zinc affect chlorophyll fluorescence in durum wheat. Int J Mol Sci 2018; 19(3): 787.

49. Tsimilli-Michael M, Strasser R. Biophysical phenomics: evalua-tion of the impact of mycorrhization with Piriformospora indica. In:V arma A, Kost G, Oelmüller R (eds) Piriformospora indica. SoilBiology. Springer, Berlin Heidelberg 2013; 173-190.

50. Bertamini M, Nedunchezhian N. Photoinhibition of photosynthesis in mature and young leaves of grapevine (Vitis vinifera L.). Plant Sci 2003; 164(4): 635-644.

51. Athar HUR, Ambreen S, Javed M, Hina M, Ashraf M. Influence of sub-lethal crude oil concentration on growth, water relations and photosynthetic capacity of maize (Zea mays L.) plants. Environ Sci Pollut Res 2016; 23(18): 18320-18331.

Figure captions

Fig 1. Shape change of leaf chlorophyll fluorescence transient curve under 5 different biofertilizer treatments.

Each curve represents the average of three independent measurements. (A) Relative chlorophyll fluorescence intensity transient curves; (B) Relative variable fluorescence intensity transient curves (V_t); (C) Normalized the relative variable fluorescence intensity transient curves (ΔV_t); (D) Relative variable fluorescence intensity transient curves of the O-J segments (W_{OJ}); (E) Normalized the relative variable fluorescence intensity transient curves of the O-J segments (ΔW_{OJ}); (F) Relative variable fluorescence intensity transient curves of the O-K segments (W_{OK}); (G) Normalized the relative variable fluorescence intensity transient curves of the O-K segments (ΔW_{OK})。No fertilizer (CK), inoculations of *Bacillus subtilis* (*Bs*, T1), combination of *Bs* and *Bacillus mucilaginosus* (*Bs*+*Bm*, T2), *Bs* and *Bacillus amyloliquefaciens* (*Bs*+*Ba*, T3), and *Bm*+*Ba* (T4).

Fig 2. Light curve (LC) of quantum yield (QY) changes under 5 biofertilizer treatments.

Data are the means of three experiments. No fertilizer (CK), inoculations of *Bacillus subtilis* (*Bs*, T1), combination of *Bs* and *Bacillus mucilaginosus* (*Bs*+*Bm*, T2), *Bs* and *Bacillus amyloliquefaciens* (*Bs*+*Ba*, T3), and *Bm*+*Ba* (T4).

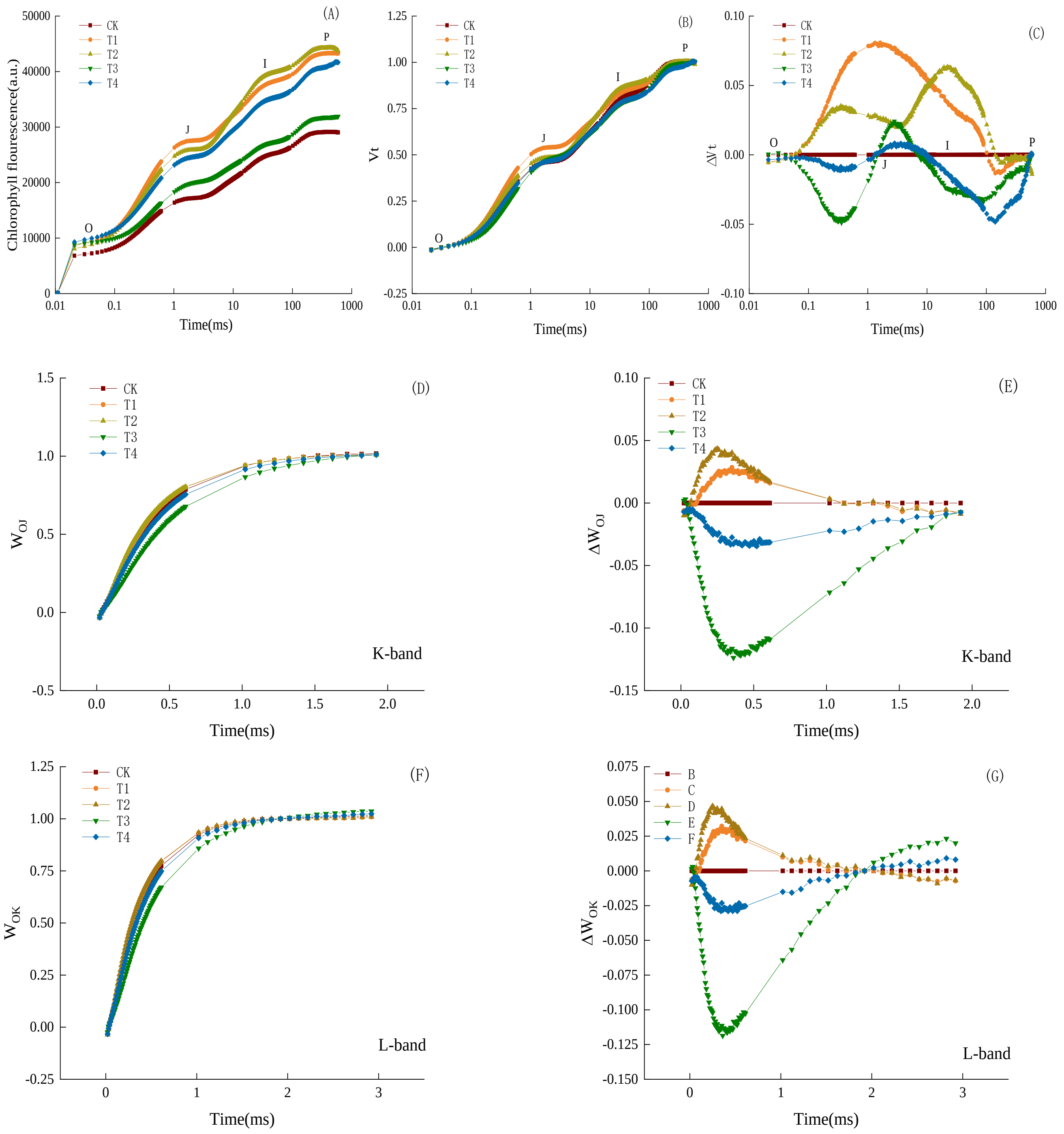
(A)
(B)
(C)
(D)
(E)
(F)
(G)
CK
T1
T2
T3
T4
B
C
D
E
F
O
J
I
P
Chlorophyll flourescence(a.u.)
Vt
ΔVt
W_{OJ}
ΔW_{OJ}
W_{OK}
ΔW_{OK}
Time(ms)
K-band
L-band

Fig 1

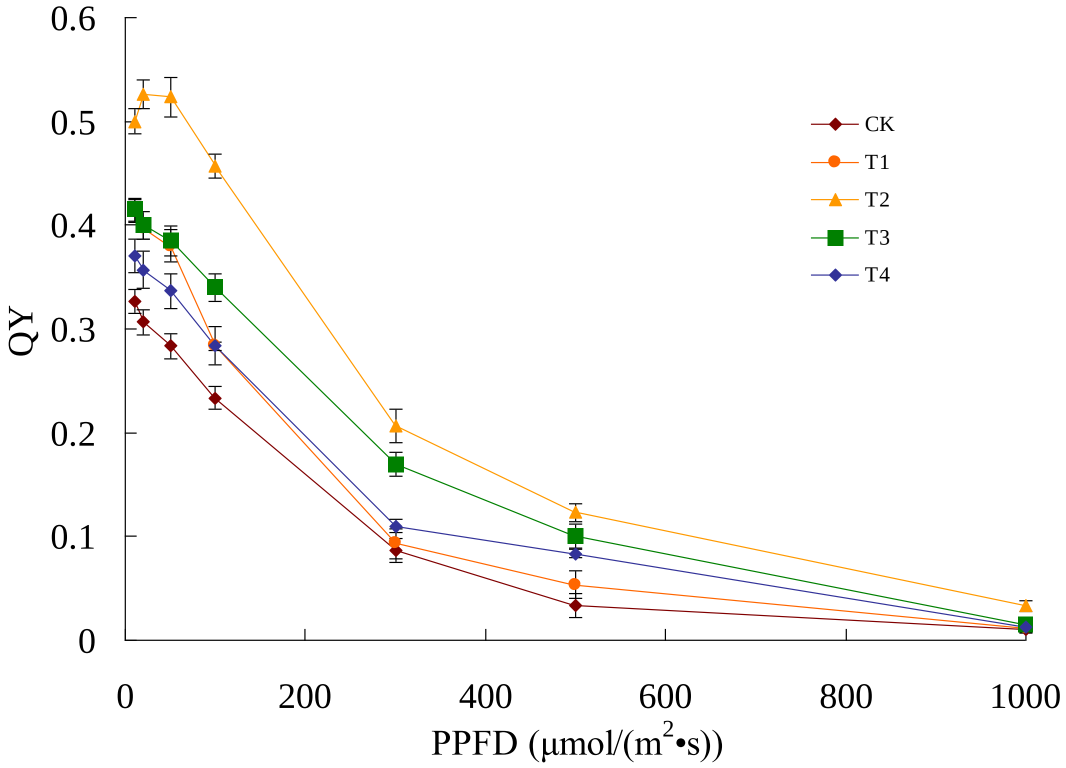

QY
0.6
0.5
0.4
0.3
0.2
0.1
0
0
200
400
600
800
1000
PPFD (μmol/(m2•s))
CK
T1
T2
T3
T4

Fig 2

Click here to access/download
Supporting Information
data.xlsx

Effects of biofertilizers on the growth, leaf physiological Indices and chlorophyll fluorescence response of spinach seedlings

Beibei Zhang[1,2*], Hui ~~Zhang[2]~~Zhang[1], Di ~~Lu[1]~~Lu[3*], Liping ~~Cheng[1]~~Cheng[3], Jiajia ~~Li[1]~~Li[3]

[1] Shaanxi Key Laboratory of Disaster Monitoring and Mechanism Simulating, College of Geography and Environment, Baoji University of Arts and Sciences, Baoji,Shaanxi, China

[2] ~~College~~ School of ~~Tourism~~ Surveying and ~~Management~~Land Information Engineering,~~,~~ ~~Pingdingshan~~ Henan Polytechnic University, ~~Pingdingshan~~Jiaozuo, Henan, ~~467000,~~ China

[3] College of Tourism and Management, Pingdingshan University, Pingdingshan, Henan, China~~.~~

~~*[2] College of computer, Baoji University of Arts and Sciences, Baoji, 721013, China.*~~

***~~Tables: 7 Figures: 2~~**

Corresponding author:

~~**Beibei Zhang**~~E-mail: zbb83101@126.com (BZ)

Current ~~Address~~Address: ~~College of Tourism and Management, Pingdingshan University, Pingdingshan, 467000,~~ Shaanxi Key Laboratory of Disaster Monitoring and Mechanism Simulating, College of Geography and Environment, Baoji University of Arts and Sciences, Baoji, China

Formatted: Superscript

Formatted: Font: Not Italic

E-mail: zbb83101@126.com

Di Lu

Address: College of Tourism and Management, Pingdingshan University, Pingdingshan, 467000, China

E-mail: ludi1128@126.com

Abstract:

–Chemcial fertilizer as the main strategy for improving the vegetable yields was excessively applied in recent years which led to progressively serious soil problems such as the soil acidification. According the situation, five different biofertilizer treatments [no fertilizer (CK), inoculations of *Bacillus subtilis* (*Bs*, , T1), combination of *Bs* and *Bacillus mucilaginosus* (*Bs*+*Bm*) (, T2), *Bs* and *Bacillus amyloliquefaciens* (*Bs*+*Ba*) (, T3), and *Bm* *and* +*Ba* ((T4))] were conducted to investigate the effect of the growth, leaf physiological indices, and chlorophyll fluorescence of spinach seedlings in the growth chamber. The growth and physiological indices of the spinach seedlings attained a maximum under the *Bs* and *Bm* T2 treatments. Under the combination of Bs and Bm T2 treatment, the ABS/RC (Absorption flux per RC), TR_0/RC (Trapping flux per RC), and ET_0/RC (Electron transport flux per RC) was

Formatted: Font: 18 pt

Formatted: Indent: First line: 0.29"

Formatted: Font: Italic

Formatted: Font: Italic

Formatted: Font: Italic

Formatted: Font: Not Italic

Formatted: Font: Not Italic

Formatted: Font: Not Italic

significantly increased, while the DI_0/RC [Dissipated energy flux per RC (at t=0)] was decreased. The OJIP curve was improved under of the inoculations of fertilizers, and the increasing range was the largest under the combination of *Bs* and *Bm* T2 treatment. The leaf light response curve (LC) was also significantly increased under the combination of Bs and BmT2 treatment. The plant growth characteristics [leaf length (LL), leaf weight (LW), plant height (PH)] were positively correlated with the J-I-P test chlorophyll fluorescence parameters [PI_{ABS} (Performance index for energy conservation from exciton to the reduction of intersystem electron acceptors), φP_0 (Maximum quantum yield of primary photochemistry), φE_0 (Quantum yield of electron transport), ψ_0 (The probability that a trapped exciton moved an electron in electron transport chain further than QA^-), TR_0/RC, and ET_0/RC] while negatively correlated with φD_0 (Quantum yield of energy dissipation) and DI_0/RC. The leaf physiological characteristics [SP (soluble protein concentrations), SC (soluble carbohydrate concentrations), Chl a (chlorophyll a), Chl b (chlorophyll b), Chl a+b, Chl a/b, and WP (water potential)] were positively correlated with the J-I-P test chlorophyll fluorescence parameters (PI_{ABS}, φP_0, φE_0, ψ_0, ABS/RC, TR_0/RC, and ET_0/RC) while negatively correlated with φD_0 and DI_0/RC. These results indicated that the combination of *Bs* and +*Bm* –inoculations promoted the growth of the spinach and improved the adaptability of the vegetable to acid soil while *Ba* inoculation didn't have any effects to plants.

Keywords: *Bacillus*; Growth; Physiological characters; Chlorophyll fluorescence

Formatted: Font: Not Italic

Formatted: Font: Not Italic

Formatted: Normal (Web)

Formatted: Bullets and Numbering

~~1.~~Introduction

Formatted: Font: Times New Roman, 18 pt

Spinach (*Spinacia oleracea L.*) is a big-leaf variety, which belonged to the spinach genus Chenopodiaceae (annual herbaceous plant), as an important leafy green vegetable its leaves and shoots that contain large quantities of bioactive compounds, minerals, and vitamins like A, B, and C and nutrients [1,2]. It can scavenge free radicals and has several medical and food applications [3]. The plant has also several antibacterial compounds and folic acid which is useful for the treatment of anemia [4]. In 2020, the global spinach planting area is about 929, 000 hectares and the production is about 31.43 million tons [5,6]. Among them, the global spinach production mainly came from China and Chinese spinach production was accounting for 91.51% of the global output [7]. So, the soil condition seriously affects the growth and yield of spinach. Recent years, fertilization as the main strategy to improve the vegetables' yield, the excessive application of chemical fertilizers has led to progressively serious soil problems such as the soil acidification, while the spinach is sensitive to it [8]. Therefore, biofertilizers have gradually replaced chemical fertilizers to improve soil quality to increase vegetable yield. Biofertilizers are also referred to as bacterial fertilizer, biological fertilizer, and microbial inoculant [9], which contains beneficial microorganisms that provide fertilization for crops through their activities.

The metabolic activities of microorganisms can improve soil fertility and crop quality, and also increase soil microorganism populations, reduce plant diseases, and enhance plant root activities [10,11]. Among them, *Bacillus* is a type of growth-promoting bacteria that can produce phytase at the rhizosphere of plants. It

has strong resistance to ultraviolet light, high salt, high acid, high heat, and radiation, with the capacity to inhibit bacteria, prevent disease, and increase production [12]. *Bacillus subtilis (Bs)* can improve the stress resistance of plants [13] and the availability of nitrogen and phosphorus in soil [14]. *Bacillus mucilaginosus (Bm)* can convert the unusable phosphorus in soil to available phosphorus for plants. Simultaneously, *Bm* can secrete substances that promote plant growth and development, such as growth hormones, gibberellin, and more [15,16]. *Bacillus amyloliquefaciens (Ba)* has a certain antagonistic effect on bacterial diseases [17].

Photosynthesis is a process through which plants convert captured light energy into biochemical energy [18]. Photosynthetic performance was found to be a very informative indicator, because of its extreme sensitivity to environment [19].Chlorophyll fluorescence measurements as one of photosynthetic performances have become a widely used method to study the functioning of the photosynthetic apparatus and are a powerful tool to study the plant’s response to different environments [20]. Through the analyses of chlorophyll fluorescence parameters, a further elucidation of the light energy absorption, utilization, transfer, and dissipation of plant chloroplast Photosystem I (PSI) and Photosystem II (PSII) processes can be obtained [21]. The chlorophyll fluorescence technique has also been widely investigated for the detection of physiological changes in plants that are caused by bacterial and fungal infections [22]. Chu et al. [23] and Bing et al. [24] revealed that there was significant correlation between chlorophyll fluorescence parameters and the degree of Verticillium wilt infection in cotton leaves. Tung et al. [25] quantitatively

analyzed the degree of infected tobacco (infected, unmanifested infection, healthy) according to Fv/Fm images, which indicated that chlorophyll fluorescence imaging technologies could detect the degree of infected tobacco.

The purpose of this research was to evaluate the variables related to growth, physiological characters and chlorophyll florescence of spinach seedlings in response to different biofertilizers under acid soil condition. The results of this study would be used to give guidance on growth, yield and soil improvement for spinach cultivation in a controlled environment.

~~2.~~Materials and Methods

~~2.1~~ Experimental materials

The spinach variety *Spinacia oleracea L.*was used as the experimental material. The *Bacillus subtilis* (*Bs*), *Bacillus mucilaginosus* (*Bm*), and *Bacillus amyloliquefaciens* (*Ba*) selected the original strains produced by Lvlong Biotecnology Co., Ltd., which contained >200 million live bacteria per gram. The fertilizer components were the *Bacillus* and its metabolites, and the rate of miscellaneous bacteria <3%.

No specific permissions were required for research locations and the studies did not involve endangered or protected species. All the authors consent for publication.

~~2.2~~ Growth conditions and experiment design

The soil came from the Qinxi demonstration garden in Taibai County whose nutrients were comprised of 33.35 g·kg^{-1} organic matter, 2.79 g·kg^{-1} total nitrogen, 1.51 g·kg^{-1} total phosphorus, and 10.75 mg·kg^{-1} available phosphorus, and a pH of 5.2.

Formatted: Font: Times New Roman, 18 pt

Formatted: Normal (Web), No bullets or numbering

Formatted: Bullets and Numbering

Formatted: Font: 16 pt, Bold, Not Italic

Formatted: Font: 14 pt, Bold, Not Italic

The experiments were carried out in some growth chambers (FytoScope FS 130-WIR, Brno, Czech) under a 12 h day/night photoperiod at temperatures of 25/15°C, respectively, a photosynthetically active radiation (PAR) of 300 µmol/(m^2·s), and humidity of 60%. According to the experiment design, the growth chambers (FytoScope FS 130-WIR) could monitor, control and adjust the temperature, PAR and humidity automatically. Five different biofertilizer treatments [no fertilizer (CK), inoculations of *Bacillus subtilis* (*Bs*, T1), combination of *Bs* and *Bacillus mucilaginosus* (*Bs+Bm*~~) (~~, T2), *Bs* and *Bacillus amyloliquefaciens* (*Bs+Ba*~~) (~~, T3), and *Bm* ~~and~~ +*Ba* (T4)] were conducted. The experimental design was a factorial scheme with 4 replicates per treatment.

The spinach seeds were soaked in deionized water for 12 h and placed in a 4°C refrigerator for 24 h to promote germination. The germinated seeds were placed on a seedling tray, covered with nutritious soil, and germinated in the dark in a 25°C incubator. Following seven days of treatment, the seedlings were transferred to pot (Ø25 cm x 20 cm high) filled with soil 18 cm deep, and 10 seedlings were planted in each pot. For this experiment, 90 kg·km^{-2} of phosphate fertilizer (potassium dihydrogen phosphate) was used as base fertilizer with biofertilizer 0.5 kg·km^{-2}.

~~2.3~~ Measurement of growth indices

For this experiment, all of the characteristics of the spinach leaves were measured at 7th day after treatments. The plant height (PH), leaf length (LL) and leaf weight (LW) were measured with a scale.

~~2.4~~ Measurement of leaf physiological indices

Formatted: Font: Italic

Formatted: Font: Italic

Formatted: Font: 14 pt, Bold, Not Italic

Formatted: Normal

Formatted: Font: 14 pt, Bold, Not Italic

Formatted: Normal

After 7 to 10 days treatment, the leaf soluble protein concentration (SP) was determined by the Coomassie brilliant blue (CBB) method at 595 nm using a DR6000 spectrophotometer [26], whereas the soluble carbohydrate concentration (SC) was determined using an anthrone method at 620 nm [27]. The leaf Chlorophyll a (Chl a) and Chlorophyll b (Chl b) concentrations were determined by Litchtenthaler [28] at 645 and 663 nm, whereas the leaf malondialdehyde (MDA) was determined by the thiobarbituric acid method [29]. The leaf water potential (WP) (ψ) was measured by dew point potentiometer (WP4, Decagon Devices, Pullman, USA).

~~2.5~~ Chlorophyll fluorescence measurements

Formatted: Font: 14 pt, Bold, Not Italic

Prior to the fluorometer measurements, the leaves were dark-adapted for 20 min and then measured using a FluorPen FP 100Max hand fluorescence meter (Photon Systems Instruments, Brno, Czech), and calculated according to the JIP-test algorithm proposed by Strasser et al. [30] (Table 1).

To compare the OJIP curve with the normalized Chlorophyll fluorescence (CF) transient curve between OJ and OK, the following formula (1), (2), (3), (4), (5) and (6) were used.

$$V_t=(F_t-F_0)/(F_m-F_0) \quad (1)$$

$$\Delta V_t=V_t^{TR}-V_t^{CK} \quad (2)$$

$$W_{OJ}=(F_t-F_0)/(F_j-F_0) \quad (3)$$

$$\Delta W_{OJ}=W_{OJ}^{TR}-W_{OJ}^{CK} \quad (4)$$

$$W_{OK}=(F_t-F_0)/(F_K-F_0) \quad (5)$$

$$\Delta W_{OK}=W_{OK}^{TR}-W_{OK}^{CK} \quad (6)$$

Table 1 Parameters of chlorophyll fluorescence

Parameters and Formula	Meaning
F_0	minimum fluorescence
F_m	maximum fluorescence
F_J	F_t at Time 2 ms
F_I	F_t at Time 30 ms
F_t	The relative fluorescence intensity at Time t
$F_v=F_m\text{-}F_0$	variable fluorescence
$nF_v=F_v/F_0$	variable to initial fluorescence ratio
$V_I=(F_J-F_0)/(F_m-F_0)$	Relative variable fluorescence at the I-step
$V_J=(F_J-F_0)/(F_m-F_0)$	Relative variable fluorescence at the J-step
M_0	Approximated initial slope (in ms^{-1}) of the fluorescence transient V= f(t)
PI_{ABS}	Performance index (potential) for energy conservation from exciton to the reduction of intersystem electron acceptors
ABS/RC	Absorption flux (of antenna Chls) per RC
$TR_0/RC=M_0(1/V_J)$	Trapping flux (leading to QA reduction) per RC
$ET_0/RC=M_0(1/V_J)\Psi o$	Electron transport flux (further than QA^-) per RC
$DI_0/RC=(ABS/RC-TR_0/RC)$	Dissipated energy flux per RC (at t = 0)
$\varphi P_0=1-F_0/F_m$	Maximum quantum yield of primary photochemistry (at t = 0)
$\varphi E_0=(1-F_0/F_m)(1-V_J)$	Quantum yield of electron transport (at t = 0)
$\Psi_0= ET_0/Tr_0=1\text{-}V_J$	The probability that a trapped exciton moved an electron in electron transport chain further than QA^-（t=0）
$\varphi D_0=F_0/F_m$	Quantum yield (at t = 0) of energy dissipation

The leaf light-response curve (LC) measurement, based on pulse modulated fluorometry (PAM) was designed using seven photosynthetic photon flux densities (PPFD) (10，20，50，100，300，500, and 1000 µmol/(m^2·s)) to acquire chlorophyll fluorescence parameter changing curves (Ft, QY) relating the rate of photosynthesis.

~~2.6~~ Statistical analysis of data

All collected data were subjected to one-way ANOVA analysis using SPSS (SPSS software version 22.0, Chicago, Illinois, USA). Differences between means

Formatted: Indent: First line: 0 ch

Formatted: Font: 14 pt, Bold, Not Italic

were compared by Bonferroni test at P<0.05. The correlations between parameters were determined using Pearson's simple correlation test function in SPSS.

3.Results

3.1 Effects of biofertilizers on growth and physiological indices of spinach seedlings

There were significant differences in plant height (PH), leaf length (LL), leaf weight (LW), and other growth parameters of the spinach seedlings under the five different treatments (F=54.37, 13.30, and 46.03, respectively; P<0.01), where the data of growth indices was maximum under the T2 treatment (Table 2). Except for Chl a+b and Chl a/b, tThere were significant differences among the treatments in the leaf physiological indices (Table 3). And the T2 treatment had the greatest impact on the leaf physiological responses. Compared with the F value, the difference in soluble sugar (SC) was the highest (F=118.35; P<0.01).

Table 2 Differences of the growth characteristics under 5 different biofertilizer treatments of spinach

Treatments	Growth characters		
	Plant height (PH, cm)	Leaf length (LL, cm)	Leaf weight (LW, g/10 plants)
None, CK	3.53±0.15 b	2.62±0.26 b	0.95±0.01 c
Bs, T1	5.23±0.09 a	4.08±0.05 a	2.08±0.02 a
Bs+Bm, T2	5.27±0.09 a	4.15±0.09 a	2.69±0.02 a
Bm+Ba, T3	5.05±0.03 a	3.73±0.18 a	1.47±0.02 b
Bs+Ba, T4	5.07±0.03 a	3.89±0.19 a	1.74±0.01 b
F value	54.37**	13.30**	46.03**

Values presented in each column of table have a mean ±standard deviation. The last portion of table refers to the F value. * P<0.05, ** P<0.01.

Formatted: Font: Times New Roman, 18 pt
Formatted: Normal (Web), No bullets or numbering
Formatted: Bullets and Numbering
Formatted: Font: 14 pt, Bold, Not Italic
Formatted: Font: 14 pt, Bold
Formatted: Font: 14 pt, Bold, Not Italic
Formatted: Indent: First line: 0"

Table 3 Differences of leaf physiological indexes under 5 different biofertilizer treatments of spinach

Treatments	Leaf physiological indexes							
	SP (mg/g FW)	SC (mg/g FW)	MDA (mg/gFW)	Chl a (mg/g FW)	Chl b (mg/g FW)	Chl a+b (mg/g FW)	Chl a/b (mg/g FW)	WP(Ψ, Mpa)
None, CK	1.83±0.26c	0.73±0.05d	5.49±0.51a	1.16±0.03e	0.36±0.03c	1.52±0.03e	3.25±0.30b	-6.57±0.25d
Bs, T1	3.31±0.20b	2.26±0.06b	1.80±0.13b	2.73±0.22b	0.58±0.06ab	3.30±0.15b	4.91±0.83a	-4.23±0.12b
Bs+Bm, T2	4.14±0.04a	3.59±0.13a	1.25±0.14b	3.23±0.04a	0.60±0.03b	3.83±0.07a	5.36±0.25a	-2.79±0.22a
Bm+Ba, T3	2.37±0.08c	1.59±0.11b	4.82±0.31a	1.70±0.07d	0.40±0.01c	2.11±0.07d	4.21±0.09ab	-5.53±0.26c
Bs+Ba, T4	3.28±0.22b	2.23±0.11c	2.09±0.15b	2.29±0.11c	0.47±0.02bc	2.75±0.09c	4.91±0.43a	-4.79±0.12b
F value	24.48**	118.35**	44.95**	50.63**	8.80**	118.34**	203.88**	48.72**

Values presented in each column of table have a mean ±standard deviation. The last portion of table refers to the F value. * P<0.05, ** P<0.01. SP: soluble protein concentrations, SC: soluble carbohydrate concentrations, MDA: malonaldehyde concentrations, Chl: chlorophyll, WP: water potential, FW: fresh weight.

~~3.2~~ Effect of biofertilizers on chlorophyll fluorescence parameters of spinach seedlings

According to the values of the basic fluorescence parameters (Table 4), the F_0 value of the T2 treatment was the highest, which was 41.29% higher than that of CK. Further, the F_m value of the T2 treatment was 17751 higher than that of CK, and there was a significant difference in F_V value under the five treatments. The relative variable fluorescence value (V_I) at 30 ms was highest under the T2 treatment. In terms of the F_v/F_0 value, the T2 treatment remained the largest. Compared with the F value, the difference of F_0 was the largest (F=112.57; P < 0.01).

Table 4 Differences of leaf chlorophyll fluorescence intensity under 5 different biofertilizer treatments of spinach

Formatted: Font: Times New Roman, 10.5 pt
Formatted: Font: Times New Roman, 10.5 pt
Formatted: Indent: First line: 0"
Formatted: Font: Times New Roman, 10.5 pt
Formatted: Font: Times New Roman, 10.5 pt
Formatted: Font: Times New Roman, 10.5 pt
Formatted: Font: Times New Roman, 10.5 pt
Formatted: Font: Times New Roman, 10.5 pt
Formatted: Font: Times New Roman, 10.5 pt
Formatted: Font: Times New Roman, 10.5 pt
Formatted: Font: Times New Roman, 10.5 pt
Formatted: Font: Times New Roman, 10.5 pt
Formatted: Font: 10.5 pt
Formatted: Font: 14 pt, Bold, Not Italic

Treatments	Leaf chlrophyll fluorescence intensity (relative unit)					
	F_0	F_m	F_v	V_J	V_I	$F_v/F_0(nF_v)$
None, CK	7087±188b	28967±1440c	21880±1628c	0.46±0.06a	0.76±0.01bc	4.07±0.03b
Bs, T1	9233±568ab	43142±1342ab	33909±1286ab	0.54±0.03ab	0.82±0.01ab	3.67±0.11a
Bs+Bm, T2	10013±10ab	46718±1126a	36705±1136a	0.47±0.00ab	0.85±0.02a	4.08±0.11a
Bm+Ba, T3	9071±108ab	31861±131bc	22790±23bc	0.47±0.01b	0.76±0.00c	2.51±0.41b
Bs+Ba, T4	9786±38a	41549±1544ab	31763±1506abc	0.46±0.00ab	0.77±0.01bc	3.25±0.24a
F value	112.57**	31.13**	24.01**	2.48	3.85*	8.3**

The values presented in each column of table have a mean ±standard deviation. The last part of table refers to the F value. * P<0.05, ** P<0.01.

Table 5 Differences of J-I-P test parameters under 5 different biofertilizer treatments of spinach leaf

Treatments	J-I-P test parameters (relative unit)					
	M_0	PI_{ABS}	ABS/RC	TR_0/RC	ET_0/RC	DI_0/RC
None, CK	0.91±0.03ab	0.38±0.29c	2.20±0.08ab	1.37±0.06ab	0.46±0.04b	0.82±0.06a
Bs, T1	1.08±0.07a	1.24±0.21ab	2.57±0.03a	2.02±0.01ab	0.94±0.03a	0.55±0.02c
Bs+Bm, T2	1.00±0.02a	1.70±0.23a	2.59±0.03a	2.08±0.04a	1.07±0.01a	0.51±0.01b
Bm+Ba, T3	0.67±0.01ab	1.41±0.01bc	2.01±0.00b	1.43±0.02b	0.76±0.01ab	0.57±0.01ab
Bs+Ba, T4	0.83±001b	1.61±0.03bc	2.35±0.08ab	1.80±0.01ab	0.96±0.01ab	0.55±0.00bc
F value	7.66**	4.09*	2.88	3.97*	2.04	8.16**

The values presented in each column of table have a mean ±standard deviation. The last part of table refers to the F value. * P<0.05, ** P<0.01.

There were significant differences between the M_0 and PI_{ABS} values under the five different treatments (F=7.66 and 4.09, respectively; P<0.05) (Table 5). The T2 treatment had the greatest effect on the specific activity of each reaction center (RC), and there was a significant difference between the TR_0/RC and DI_0/RC. However, there was no significant difference in the ABS/RC and ET_0/RC between the treatments. The quantum yield (φP_0), efficiency (φE_0), and ψ_0 values were highest

Formatted: Indent: First line: 0"

Formatted: Indent: First line: 0"

Formatted: Font: 10.5 pt

under the T2 treatment, and with the exception of ψ_0, there were significant differences (Table 6) (P<0.01). On the contrary, the value of φD_0 was lowest under the T2 treatment.

Table 6 Differences of leaf chlorophyll fluorescence yield and efficiency under 5 different biofertilizer treatments of spinach

Treatments	Yield and efficiency			
	$\varphi P_0(F_v/F_m)$	φE_0	Ψ_0	φD_0
None, CK	0.62±0.01c	0.22±0.01ab	0.34±0.01ab	0.37±0.01ab
Bs, T1	0.78±0.01ab	0.37±0.01a	0.47±0.01a	0.21±0.00c
Bs+Bm, T2	0.80±0.00a	0.41±0.06b	0.52±0.05a	0.19±0.00bc
Bm+Ba, T3	0.72±0.0bc	0.38±0.03ab	0.53±0.02b	0.29±0.03a
Bs+Ba, T4	0.76±0.01ab	0.41±0.00ab	0.54±0.00ab	0.24±0.01bc
F value	6.03**	2.48	2.71	6.02**

The values presented in each column of table have a mean ±standard deviation. The last part of table refers to the F value. * P<0.05, ** P<0.01.

Formatted: Indent: First line: 0"

~~3.3~~ Effect of biofertilizers on transient analysis of prompt fluorescence OJIP of spinach seedlings

Formatted: Font: 14 pt, Bold, Not Italic

The transient curve of OJIP was shown in Fig. 1 and the OJIP curves of the five treatments were similar to those reported by Strasser et al. [31]. In Fig. 1(A), compared with CK, the fluorescence trend of other treatments gradually increased. Among them, the fluorescence value of the T2 treatment was the largest, which signified that it had a greater effect on the photosynthetic chemical rate of the leaves. As shown in Fig. 1(B), the difference of relative variable fluorescence (Vt) at point J (2 ms) was the largest. This was better illustrated in Fig. 1(C) ΔV_t. Over time the difference between the treatments increased initially, then decreased, and the

difference of the initial slope during the O-J stage curve was maximized. It can be seen in Figs 1 D and E, while there was an obvious positive K band under the T1 and T2 treatments, and a negative growth trend under the T3 and T4 treatments. The transient double normalization of OJIP between F_0 and F_K and the difference of double normalization between the treatments and the control group were regarded as the L band. As shown in Figs 1 F and G, the W_{OK} value of the T2 treatment was higher, and there was a significant higher positive L band.

Effect of biofertilizers on the difference of the Light response Curve (LC) of the spinach seedlings

Formatted: Font: 14 pt, Bold, Not Italic

In Fig 2, compared with the five treatments, the quantum yield (QY) of leaves under the T2 treatment attained a maximum at about 20 μmol/(m^2·s) PPFD, which indicated that the light compensation point (LCP) was around 20 μmol/(m^2·s) PPFD. The maximum value of the other treatments was before 10 μmol/(m^2·s) PPFD, indicating that the LCP was earlier than 10 μmol/(m^2·s) PPFD.

Correlations between growth, leaf physiological indices and chlorophyll fluorescence

Formatted: Font: 14 pt, Bold, Not Italic

Through correlation analyses, the indices of growth, physiology, and chlorophyll fluorescence of leaves were quantified. Plant growth indices (LL, LW, PH) were positively correlated with chlorophyll fluorescence parameters (PI_{ABS}, φP_0, φE_0, ψ_0, TR_0/RC, ET_0/RC), while which was negatively correlated with φD_0 and DI_0/RC. The physiological indices of the leaves (SP, SC, Chl a, Chl b, Chl a+b, Chl a/b, and WP) were positively correlated with PIABS, φP_0, φE_0, ψ_0, ABS/RC, TR_0/RC and ET_0/RC,

and negatively correlated with φD_0 and DI_0/RC. The leaf MDA was significantly positively correlated with φD_0 and DI_0/RC, and positively correlated with other chlorophyll fluorescence parameters (Table 7).

Table 7 Correlations between the leaf physiological characteristics and chlorophyll fluorescence parameters

Index	LW	LL	PH	SP	SC	Chl a	Chl b	Chl a+b	Chl a/b	MDA	WP
PI_{ABS}	0.75**	0.84**	0.92**	0.76**	0.81**	0.73**	0.55*	0.73**	0.68**	-0.71**	0.75**
φP_0	0.90**	0.89**	0.91**	0.89**	0.90**	0.93**	0.80**	0.93**	0.74**	-0.91**	0.90**
φE_0	0.58*	0.78**	0.89**	0.60*	0.65**	0.56*	0.38	0.55*	0.58*	-0.55*	0.58*
Ψ_0	0.74**	0.86**	0.95**	0.75**	0.79**	0.74**	0.56*	0.74**	0.68**	-0.72**	0.74**
φD_0	-0.90**	-0.89**	-0.91**	-0.89**	-0.90**	-0.93**	-0.80**	-0.93**	-0.74**	0.91**	-0.90**
ABS/RC	0.77**	0.51	0.40	0.78**	0.73**	0.83**	0.80**	0.84**	0.54*	-0.85**	0.78**
TR_0/RC	0.90**	0.74**	0.69**	0.90**	0.88**	0.94**	0.86**	0.96**	0.68**	-0.95**	0.90**
ET_0/RC	0.89**	0.88**	0.90**	0.91**	0.92**	0.91**	0.76**	0.92**	0.75**	-0.91**	0.90**
DI_0/RC	-0.80**	-0.90**	-0.97**	-0.78**	-0.82**	-0.80**	-0.65**	-0.81**	-0.70**	0.76**	-0.79**

* P<0.05** P<0.01. LL: Leaf length, LW: Leaf weight, PH: Plant height, SP: Soluble protein concentrations, SC: Soluble carbohydrate concentrations, MDA: malonaldehyde concentrations, Chl: chlorophyll, WP: Water potential.

~~4.1~~Discussion

~~4.1~~Physiological indices of growth and leaves

In terms of plant phenology and biological characteristics, plant growth, leaf chlorophyll concentration, and other physiological indices were critical influencing

Formatted: Indent: First line: 0"

Formatted: Font: Times New Roman, 18 pt

Formatted: Font: Times New Roman, 18 pt

Formatted: Bullets and Numbering

Formatted: Font: Times New Roman, 18 pt

Formatted: Normal (Web), No bullets or numbering

Formatted: Font: 14 pt, Bold, Not Italic

Formatted: Bullets and Numbering

Formatted: Font: 14 pt, Bold

Formatted: Font: 14 pt, Bold, Not Italic

factors [32]. The results of Yan et al. [33] revealed that the application of biofertilizer could effectively improve the plant height of pakchoi. In this study, the inoculations of biofertilizer effectively improved the plant height, leaf length, and leaf weight of spinach~~,~~ . ~~among which t~~The growth status of plants with the inoculations of *Bs* ~~and~~ or *Bm* was more dominant ~~while which~~and it was similar to the results of Samia et al. [34] ~~while the inoculation of *Ba* didn't have more effects. The reason for the analysis may be that *Ba* mainly targets the repair effect of plant facing to bacterial and fungal infections, while the plants used in the experiment were not infected, so the promoting effect on plant growth is not significant.~~.

The leaf soluble protein (SP), soluble carbohydrate (SC), and water potential (WP) were closely related to plant metabolism. Chlorophyll (Chl) was an important substance in plant photosynthesis, and malondialdehyde (MDA) can reflect the degree of membrane lipid peroxidation [35]. In this research, When *Bs* and *Bm* (T2) were inoculated together, the leaf physiological indices of spinach seedling (SP, SC, Chl a, Chl b, Chl a+b, Chl a/b, and WP) were significantly enhanced. ~~biofertilizer Bs and Bm significantly enhanced the leaf physiological indices of spinach seedling (SP, SC, Chl a, Chl b, Chl a+b, Chl a/b, and WP).~~ Wang et al. [36] found that the application of biofertilizer could effectively increase the chlorophyll concentration of winter wheat at the different growth stages. The application of phosphate fertilizer can promote the synthesis of chlorophyll in leaves [37].

For this study, the leaf chlorophyll concentration treated with the combination of *Bs* ~~and~~ +*Bm* treatment (T2) was the highest, whereas when *Bs* or *Bm* were paired with

Formatted: Font: Italic

Formatted: Font: Italic

Formatted: Font: Not Italic

Formatted: Font: Not Italic

Formatted: Font: Italic

Formatted: Font: Italic

Ba there was less chlorophyll. It might be possibly explained that it could convert ineffective nitrogen or phosphorus into available nitrogen or phosphorus in the soil and improved the increase in N fixation and P solubilization of the plants after the application of –*Bs* and or *Bm*-. Thus promoting the synthesis of ATP (Adenosine triphosphate) and NADPH (nicotinamide adenine dinucleotide phosphate) in leaves then promoting the growth. Likewise, after inoculation it also could affect the regulation of phytohormone biosynthesis pathways; modulate ethylene levels in plants and the launch of host plants' systemic tolerance [38]. converted ineffective phosphorus into available phosphorus in the soil, which improved the availability of phosphorus and enhanced the plant absorbing ability, thus promoting the synthesis of ATP (Adenosine triphosphate) and NADPH (nicotinamide adenine dinucleotide phosphate) in leaves then promoting the growth. The inoculation of *Ba* didn't have more effects. The reason for the analysis may be that *Ba* mainly targets the repair effect of plant facing to bacterial and fungal infections, and the plants used in the experiment were not infected, so the promoting effect on plant growth is not significant. When plants were under stress, the MDA concentration was typically an important indicator of membrane lipid peroxidation, which reflected the harmful effects of stress on plant cells and tissues [3839]. In this study, the concentration of MDA in leaves treated with biofertilizer was decreased which mean the plant was in a suitable environment.

4.2Leaf chlorophyll fluorescence

Formatted: Font: Italic

Formatted: Font: 14 pt, Bold, Not Italic

Formatted: Bullets and Numbering

In this research, the values of F_0 and Fm were enhanced, which indicated that the *Bs* and *Bm* promoted an increase in the size of leaf PSII antenna and a decrease in the non-radiative dissipation of chlorophyll in PSII antenna, thus increasing the capacity of leaves to capture light energy [[3940]. We observed higher Fv and F_v/F_0 (nF_v) values under the combination of *Bs* and +*Bm* treatment, which signified an increase in the efficiency of supplying electrons to PSII reaction centre (RCS) and the photosynthetic quantum conversion rate of PSII RCS. This translated to less energy being used for non-photochemical dissipation in the dark adaptation process [4041].

In this experiment, the ABS/RC values of the leaves increased, indicating that after inculcating *Bs* and +*Bm* the size of active RCS was increased, which led to a higher number of active PSII reaction centers and enhanced dark accumulation [20]. Under the action of the *Bs* and +*Bm*, the TR_0/RC value increased, which reflected the higher electron capture rate of RC, where more QA (the primary electron acceptor in Photosystem II) was converted to QA^-. This resulted in an increase in the electron transfer energy (ET_0/RC), thus reducing the energy dissipated by non-photochemical activities (DI_0/RC) [4142,4243].

The φP_0 (Fv/Fm) represented the maximum quantum yield of PS Ⅱ, and the ψ_0 value reflected the electron transfer efficiency, from QA^- to QB, whereas φE_0 reflected the quantum yield of electron transport. The increase of φP_0, ψ_0, and φE_0 indicated that the combination of *Bs* and +*Bm* promoted the redox reaction following QA, which resulted in an increase of the electron transfer rate between QA^- and QB [4344].

Oukarroum et al. [39] regarded the double normalization of the OJIP transient between the peak value of 0.05 to 2 ms, and the difference of double normalization between the treatment and the control group as the K-band. The change of the chlorophyll fluorescence curve was closely related to the physiological morphology of plants [~~44~~45]. Changes in the O-J segment were related to an increased number of inactive reaction centers, or the energy transfer from the LHC II (Light-harvesting Complex) to PS II RCS [~~45~~46]. The K and L bands reflected the connection between the S state of the PSII oxygen evolution complex (OEC) and PS II unit, as well as the energy connection between the PS II units [~~46~~47,~~47~~48].

An increase in the J-I segment could reflect a decrease in the relative number of active PQ (plastoquinone) molecules that was reduced by each active RCS of PSII [~~47~~48]. Further, changes in the I-P segment were closely related to the pool of electron receptors (ferredoxin and NADPH) at the end of the PSI, signifying the kinetic flow rate to the electron receptors at the end of the PSI [~~48~~49]. In this experiment, the OJIP transient curves of the spinach seedlings were affected by biofertilizer (Fig.1).

Under the ~~combination of *Bs* and *Bm*~~T2 treatment, the relative fluorescence intensity of chlorophyll in the O-J segment exhibited a larger value, indicating that the population of active PSII centers decreased, whereas the QA^- accumulated massively. Fig.1 (E and G) showed that there were obvious K bands and positive L bands under the ~~combination of *Bs* and *Bm*~~T2 treatment, which indicated that the PSII had an inhibitory effect on the OEC. This resulted in a weakening of the connectivity

between PSII and OEC and a decreased energy connection between the PSII units. This might due to the variable light and ventilation that was present at different locations in the incubator, which might have led to PSI receptor side damage and chlorophyll protein denaturation in some plant leaves [~~49~~50]. In addition, the fluorescence of J-I and I-P segments remained large under the ~~combination of *Bs* and *Bm*~~T2 treatment, which indicated that the relative number of PQ decreased, while there was an increase in the pool of electron receptors (ferredoxin and NADPH) at the end of PSI, which led to a higher kinetic flow rate to the electron receptors at the end of PSI. Combined with PI_{ABS}, the higher PI_{ABS} values under combination of *Bs* and *Bm* treatment indicated increases in the density of active PSII centers, the efficiency of photoreactions, and efficiency of biochemical dark redox reactions, as well as the production and utilization of NADPH [~~50~~51].

~~5.~~Conclusions

In this study, the role of different biofertilizers was analyzed in spinach seedling. It was observed that the inoculations of biofertilizers *Bs* ~~and~~ or *Bm* stabilized the p~~al~~lant cell membrane and increased the rate of photosynthesis which led toward the improvement of plant growth. Acid soil reduced the growth of plant and increased the amount of ROS production which disrupted the plant metabolism. The application of biofertilizers triggered osmotic adjustment, maintained the production of antioxidants, improved the leaf chlorophyll fluorescence and promoted the growth in plants. The ~~combined inoculations of *Bs* and *Bm*~~T2 treatment significantly ($p<0.05$) increased the

Formatted: Font: Times New Roman, 18 pt

Formatted: Normal (Web), No bullets or numbering

Formatted: Bullets and Numbering

growth, physiological index and leaf chlorophyll fluorescence of spinach. It could be concluded that combined inoculations of *Bs*~~ and ~~+*Bm* was a better strategy for reducing the negative effects of acid soil on spinach. Due to limited experimental conditions, the experiment was only analyzed at 300 μmol/(m^2•s) light conditions without designing the multiple light conditions. In the next experiment, the impact of biofertilizer inoculation on spinach under multiple light conditions will be explored. Likewise, ~~F~~future research also should endeavor to analyze the utilization of mineral elements in the soil, prevent soil acidification caused by the excessive accumulation of mineral elements and spinach quality to control the fertilization concentrations of microorganisms, so as to provide an improved theoretical basis for spinach production and obtained the great economic benefits.

Formatted: Superscript

Declarations

- **Ethics approval and consent to participate**

The research passed the ethics approval and it was consent to participate.

- **Consent to publish**

All the authors consent for publication.

- **Availability of data and materials**

The data and materials are available.

- **Competing interests**

All the authors declare no competing interests.

~~- **Funding**~~

This work was supported by the National Natural Science Foundation of China (NSFC, No. 41601016, 41771036), Special Support Program for High-Level Personnel Recruitment (Youth Top-Talent) of Shaanxi province, National Natural Science Foundation of Shaanxi province (No. 2015JM4136), Doctoral research start-up fee of Pingdingshan University (PXY-BSQD-2022039), Key projects of Shaanxi Provincial Department of Education(20JS011).

- Authors' Contributions

D. L. and J. J. L. prepared the materials. B. B. Z designed the study. L. P. C. did the data analysis. B. B. Z and H. Z. wrote the main manuscript text. All authors discussed the results and commented on the paper.

- Acknowledgements

The authors are grateful to Jing Qi, Jiaxuan Wang, Jiayi Han and Xiaoying Hu for assistance with the data measuring.

- Data availability

All data are available upon request by contact with the corresponding author. The minimal data set as a Supporting Information file uploaded.

References

1. Xu CP, Leskovar DI. Effects of A. nodosum seaweed extracts on spinach growth, physiology and nutrition value under drought stress. Sci Hortic 2015; 183: 39-47.
2. Roberts JL, Moreau R. Functional properties of spinach (Spinacia oleracea L.) phytochemicals and bioactives. Food funct 2016; 7(8): 3337-3353.

3. Bunea A, Andjelkovic M, Socaciu C, Bobis O, Neacsu M, Verhé R, Camp JV. Total and individual carotenoids and phenolic acids content in fresh, refrigerated and processed spinach (Spinacia oleracea L.). Food Chem 2008; 108 (2): 649-656.

4. Atti AR, Palmer K, Volpato S, Zuliani G, Winblad B, Fratiglioni L. Anaemia increases the risk of dementia in cognitively intact elderly. Neurobiol Aging 2006; 27 (2): 278-284.

6. Wang M. Analysis of spinach planting area, output and price in the world and China in 2020. Industrial Information Network, 2020-04-08: https://www.chyxx.com/industry/202104/943941.html.

7. Awika HO, Solorzano J, Cholula U, Shi AR, Enciso J, Avila CA. Prediction modeling for yield and water-use efficiency in spinach using remote sensing via an unmanned aerial system. Smart Agr Tech 2021; 1: 100006.

8. Dawson CJ, Hilton J. Fertilizer availability in a resource-limited world: production and recycling of nitrogen and phosphorus. Food Policy 2012; 36: 14-22.

9. Osman M, El-Sheekh MM, El-Naggar AH, Gheda SH. Effect of two species of cyanobacteria as biofertilizers on some metabolic activities, growth, and yield of pea plant. Biol Fert Soils 2010; 46(8): 861-875.

10. Cao EH, Hou XW, Li GY, et al. Effect of combination bacteria on soil physicochemical properties and soil microbial activity by pot tomato experiments. Ecology and Environmental Sciences 2011; 20(5): 875-880.

11. Dinesh R, Srinivasan V, Hamza S, Manjusha A. Short-term incorporation of organic manures and biofertilizers influences biochemical and microbial characteristics of soils under an annual crop [Turmeric (*Curcuma longa L.*)]. Bioresource Techno 2010; 101(12): 4697-4702.
12. Guérout-Fleury AM, Shazand K, Frandsen N. Antibiotic-resistance cassettes for *Bacillus subtilis*. Gene 1995; 167(1-2): 335-336.
13. Pliego C, Ramos C. Screening for candidate bacterial biocontrol agents against soilborne fungal plant pathogens. Plant Soil 2010; 340(1): 505-520.
14. Idriss EE, Makarewicz O, Farouk A, Rosner K, Greiner R, Bochow H, Richter T, Borriss R. Extracellular phytase activity of Bacillus amyloliquefaciens FZB45 contributes to its plant-growth-promoting effect. Microbiology 2002; 148(7): 2097-2109.
15. Liu SN,Tang WZ, Yang F Meng J, Li XZ. Influence of biochar application on potassium-solubilising *Bacillus mucilaginosus* as potential biofertilizer. Prep Biochem Biotech 2016; 1: 32-37.
16. ZhaoY, Zhang XB, GuoW. Physiological and biochemical characteristics and capacities of potassium-releasing of *Bacillus mucilaginosus* screened from different soils. Ecol Environ Sci 2009; 6: 2283-2286.
17. Arrebola E, Jacobs R, Korsten L. Iturin A is the principal inhibitor in the biocontrol activity of Bacillus amyloliquefaciens PPCB004 against postharvest fungal pathogens. J Appl Microbiol 2010; 108(2):386-395.

18. Maxwell K, Johnson GN. Chlorophyll fluorescence e a practical guide. J Exp Bot 2000; 51: 659-668.

19. Massacci A, Nabiev SM, Pietrosanti L, Nematov SK, Chernikova TN, Thor K, Leipner J. Response of the photosynthetic apparatus of cotton (*Gossypium hirsutum*) to the onset of drought stress under field conditions studied by gas-exchange analysis and chlorophyll fluorescence imaging. Plant Physiol Bioch 2008; 46: 189-195.

20. Mehta P, Jajoo A, Mathur S, Bharti S. Chlorophyll a fluorescence study revealing effects of high salt stress on Photosystem II in wheat leaves. Plant Physiol Bioch 2010; 48: 16-20.

21. Li PM, Gao HY, Reto JS. Application of the fast chlorophyll fluorescence induction dynamics analysis in photosynthesis study. J Pla Physiol Mol Biol 2005; 31(6): 559-566.

22. Zaidi A, Ahmad E, Khan MS, Saif S, Rizvi A. Role of plant growth promoting rhizobacteria in sustainable production of vegetables: Current perspective. Sci Hortic 2015; 193: 231-239.

23. Chu Z, Xin W, Li Y, Yu H, Li J, Lu Y, Li H, Bo O. Genomic Organization, Phylogenetic and Expression Analysis of the B-BOX Gene Family in Tomato. Front Plant Sci 2016; 10(7): 1522.

24. Bing C, Wang KR, Li SK, Xiao CH, Wang K. Monitoring Cotton Field with Suspected Verticillium wilt Using Satellite Remote Sensing with TM Satellite Image as an Example. Acta Agron Sinica 2012; 38(1):129-139.

25. Tung J, Goodwin PH, Hsiang T. Chlorophyll fluorescence for quantification of fungal foliar infection and assessment of the effectiveness of an induced systemic resistance activator. Eur J Plant Pathol 2013; 136(2): 301-315.

26. Bradford MM. Arapid and sensitive method for the quantitation of microgram quantities of protein utilizing the principle of proteindye binding. Anal Biochem 1976; 72: 248-254.

27. Jermyn MA. Increasing the sensitivity of the anthrone method for carbohydrate. Anal Biochem 1975; 68(1): 332-335.

28. Litchtenthaler HK, Wellburn AR. Determinations of total carotenoids and chlorophylls a and b of leaf extracts in different solvents. Biochem Soc T 1983; 11(5): 591-592.

29. Draper HH, Squires EJ, Mahmoodi H,Wu J, Agarwal S, Haley M. A Comparative evaluation of thiobarbituric acid methods for the determination of malondialdehyde in biological materials. Free Radical Bio Med 1993; 15(4): 353-363.

30. Strasser RJ, Tsimilli-Michael M, Qiang S, Goltsev V. Simultaneous in vivo recording of prompt and delayed fluorescence and 820-nm reflection changes during drying and after rehydration of the resurrection plant Haberlea rhodopensis. BBA-Biomembranes 2010; 1797: 1313-1326.

31. Strasser RJ, Tsimilli-Michael M, Srivastava A. Analysis of the chlorophyll a fluorescence transient. In: Papageorgiou, G.C., Govindjee (Eds.), Chlorophyll

Fluorescence: a Signature of Photosynthesis. Kluwer Academic Publishers Press, Netherlands 2004; 321-362.

32. Bennie J, Davies TW, CruseD, et al. Ecological effects of artificial light at night on wild plants. J Eco 2016; 104(3): 611-620.

33. Yan DY, He XY, Li YG. Effect of compound microbial fertilizer on the production of brassica chinensis. J Anhui Agri 2017; 45(14): 33-34.

34. Sami AA, Salwa MA. Application of trichoderma Harzianum T22 as a biofertilizer poteneial in maize growth. J Plant Nutr 2014; 37(1): 30-49.

35. Xi ZM, Wang ZZ, Fang YL, Hu ZY, Hu Y, Deng MM, Zhang ZW. Effects of 24- epibrassinolide on antioxidation defense and osmoregulation systems of young grapevines (V. vinifera L.) under chilling stress. Plant Growth Regul 2013; 71: 57-65.

36. Wang JW, Zhao JJ, Li GL. Effects of new compound microbial fertilizer on physiological indexes of winter wheat. J South Agri 2018; 49(10): 1953-1958.

37. Li FM, Song QH, Liu HS, Li FR, Liu XL. Effects of pre-sowing irrigation and phosphorus application on water use and yield of spring wheat under semi-arid conditions. Agr Water Manage 2001; 49(3): 173-183.

38. Awan ZA, Shoaib A. Combating early blight infection by employing Bacillus subtilis in combination with plant fertilizers. Curr Plant Biol 2019; 20: 100125.

39~~8~~. Han YY, Fan SX, Qiao Z, Wang Y. Effect of heat stress on the MDA, proline and soluble sugar content in leaf lettuce seedlings. Agri Sci 2013; 4(5): 112-115.

~~39~~40. Oukarroum A, Madidi SDE, Schansker G. Probing the responses of barley cultivars (Hordeum vulgare L.) by chlorophyll a fluorescence OLKJIP under drought stress and re-watering. Environ Exp Bot 2007; 60(3): 438-446.

~~40~~41. Spoustova P, Synkova H, Valcke R. Chlorophyll a fluorescence as a tool for a study of the Potato virus Y effects on photosynthesis of nontransgenic and transgenic Pssuipt tobacco. Photosynthetica 2013; 51(2): 191-201.

~~41~~42. Yiotis C, Manetas Y. Sinks for photosynthetic electron flow in green petioles and pedicels of Zantedeschia aethiopica: evidence for innately high photorespiration and cyclic electron flow rates. Planta 2010; 232: 523-531.

~~42~~43. Tang YP, Sun X, Wen T, Liu MJ, Yang MY, Chen XF. Implications of terminal oxidase function in regulation of salicylic acid on soybean seedling photosynthetic performance under water stress. Plant Physiol Bioch 2017; 112: 19-28.

~~43~~44. Lebkuecher JG, Haldeman KA, Harris CE, Holz S, Joudah SA, Milton DA. Development of photosystem II activity during irradiance of etiolated Helianthus (Asteraceae) seedlings. Am J Bot 1999; 86(8): 1087-1092.

~~44~~45. Malaspina P, Giordani P, Pastorino G, Modenesi P, Mariotti MG. Interaction of sea salt and atmospheric pollution alters the OJIP fluorescence transient in the lichen Pseudevernia furfuracea (L.) Zopf. Ecol Indic 2015; 50, 251-257.

~~45~~46. Tomar RS, JajooA. A quick investigation of the detrimental effectsof environmental pollutant polycyclic aromatic hydrocarbon fluo-ranthene on the

photosynthetic efficiency of wheat (Triticum aestivum). Ecotoxicology 2013; 22: 1313-1318.

4647. Dąbrowski P. BaczewskaAH, Pawluśkiewicz B. Prompt chlorophyll a fluorescence as a rapid tool for diagnostic changes in PSII structure inhibited by salt stress in Perennial ryegrass. J Photoch Photobio B 2016; 157: 22-31.

4748. Paunov M, Koleva L, Vassilev A. Effects of different metals on photosynthesis: cadmium and zinc affect chlorophyll fluorescence in durum wheat. Int J Mol Sci 2018; 19(3): 787.

4849. Tsimilli-Michael M, Strasser R. Biophysical phenomics: evalua-tion of the impact of mycorrhization with Piriformospora indica. In:V arma A, Kost G, Oelmüller R (eds) Piriformospora indica. SoilBiology. Springer, Berlin Heidelberg 2013; 173-190.

4950. Bertamini M, Nedunchezhian N. Photoinhibition of photosynthesis in mature and young leaves of grapevine (Vitis vinifera L.). Plant Sci 2003; 164(4): 635-644.

5051. Athar HUR, Ambreen S, Javed M, Hina M, Ashraf M. Influence of sub-lethal crude oil concentration on growth, water relations and photosynthetic capacity of maize (Zea mays L.) plants. Environ Sci Pollut Res 2016; 23(18): 18320-18331.

Formatted: Font: 12 pt

Formatted: Indent: Left: 0", Hanging: 2 ch

Tables

Table 1 Parameters of chlorophyll fluorescence

Parameters and Formula	Meaning
F_0	minimum fluorescence
F_m	maximum fluorescence
F_J	F_t at Time 2 ms
F_I	F_t at Time 30 ms
F_t	The relative fluorescence intensity at Time t
$F_v=F_m-F_0$	variable fluorescence
$nF_v=F_v/F_0$	variable to initial fluorescence ratio
$V_I=(F_J-F_0)/(F_m-F_0)$	Relative variable fluorescence at the I-step

$V_J=(F_J-F_0)/(F_m-F_0)$	Relative variable fluorescence at the J-step
M_0	Approximated initial slope (in ms^{-1}) of the fluorescence transient V= f(t)
PI_{ABS}	Performance index (potential) for energy conservation from exciton to the reduction of intersystem electron acceptors
ABS/RC	Absorption flux (of antenna Chls) per RC
$TR_0/RC=M_0(1/V_J)$	Trapping flux (leading to QA reduction) per RC
$ET_0/RC=M_0(1/V_J)\Psi o$	Electron transport flux (further than QA^-) per RC
$DI_0/RC=(ABS/RC-TR_0/RC)$	Dissipated energy flux per RC (at t = 0)
$\varphi P_0=1-F_0/F_m$	Maximum quantum yield of primary photochemistry (at t = 0)
$\varphi E_0=(1-F_0/F_m)(1-V_J)$	Quantum yield of electron transport (at t = 0)
$\Psi_0=ET_0/Tr_0=1-V_J$	The probability that a trapped exciton moved an electron in electron transport chain further than QA^-（t=0）
$\varphi D_0=F_0/F_m$	Quantum yield (at t = 0) of energy dissipation

Table 2 Differences of the growth characteristics under 5 different biofertilizer treatments of spinach

Values presented in each column of table have a mean

Treatments	Growth characters		
	Plant height (PH, cm)	Leaf length (LL, cm)	Leaf weight (LW, g/10 plants)
CK	3.53±0.15 b	2.62±0.26 b	0.95±0.01 c
T1	5.23±0.09 a	4.08±0.05 a	2.08±0.02 a
T2	5.27±0.09 a	4.15±0.09 a	2.69±0.02 a
T3	5.05±0.03 a	3.73±0.18 a	1.47±0.02 b
T4	5.07±0.03 a	3.89±0.19 a	1.74±0.01 b
F value	54.37**	13.30**	46.03**

Formatted Table

~~±standard deviation. The last portion of table refers to the F value. * P<0.05, ** P<0.01.~~

~~Table 3 Differences of leaf physiological indexes under 5 different biofertilizer treatments of spinach~~

~~Treatments~~	~~Leaf physiological indexes~~							
	~~SP (mg/g FW)~~	~~SC (mg/g FW)~~	~~MDA (mg/gFW)~~	~~Chl a (mg/g FW)~~	~~Chl b (mg/g FW)~~	~~Chl a+b (mg/g FW)~~	[illegible] ~~(mg/g FW)~~	[illegible] ~~(Ψ, Mpa)~~
~~CK~~	~~1.83±0.26c~~	~~0.73±0.05d~~	~~5.49±0.51a~~	~~1.16±0.03e~~	~~0.36±0.03c~~	~~1.52±0.03e~~	~~3.25±0.30b~~	~~-6.57±0.25d~~
~~T1~~	~~3.31±0.20b~~	~~2.26±0.06b~~	~~1.80±0.13b~~	~~2.73±0.22b~~	~~0.58±0.06ab~~	~~3.30±0.15b~~	~~4.91±0.83a~~	~~-4.23±0.12b~~
~~T2~~	~~4.14±0.04a~~	~~3.59±0.13a~~	~~1.25±0.14b~~	~~3.23±0.04a~~	~~0.60±0.03b~~	~~3.83±0.07a~~	~~5.36±0.25a~~	~~-2.79±0.22a~~
~~T3~~	~~2.37±0.08c~~	~~1.59±0.11b~~	~~4.82±0.31a~~	~~1.70±0.07d~~	~~0.40±0.01c~~	~~2.11±0.07d~~	~~4.21±0.09ab~~	~~-5.53±0.26c~~
~~T4~~	~~3.28±0.22b~~	~~2.23±0.11c~~	~~2.09±0.15b~~	~~2.29±0.11c~~	~~0.47±0.02bc~~	~~2.75±0.09c~~	~~4.91±0.43a~~	~~-4.79±0.12b~~
~~F value~~	~~24.48**~~	~~118.35**~~	~~44.95**~~	~~50.63**~~	~~8.80**~~	~~0.12~~	~~3.34~~	~~48.72**~~

Formatted Table

Values presented in each column of table have a mean ±standard deviation. The last portion of table refers to the F value. * P<0.05, ** P<0.01. SP: soluble protein concentrations, SC: soluble carbohydrate concentrations, MDA: malonaldehyde concentrations, Chl: chlorophyll, WP: water potential, FW: fresh weight.

Table 4 Differences of leaf chlorophyll fluorescence intensity under 5 different biofertilizer treatments of spinach

Treatments	Leaf chlrophyll fluorescence intensity (relative unit)					
	F_0	F_m	F_v	V_J	V_I	F_v/F_0(mF_v)
CK	7087±188b	28967±1440c	21880±1628c	0.46±0.06a	0.76±0.01bc	4.07±0.03b
T1	9233±568ab	43142±1342ab	33909±1286ab	0.54±0.03ab	0.82±0.01ab	3.67±0.11a
T2	10013±10ab	46718±1126a	36705±1136a	0.47±0.00ab	0.85±0.02a	4.08±0.11a
T3	9071±108ab	31861±131bc	22790±23bc	0.47±0.01b	0.76±0.00c	2.51±0.41b
T4	9786±38a	41549±1544ab	31763±1506abc	0.46±0.00ab	0.77±0.01bc	3.25±0.24a
F value	112.57**	31.13**	24.01**	2.48	3.85*	8.3**

Formatted Table

The values presented in each column of table have a mean ±standard deviation. The last part of table refers to the F value. * P<0.05, ** P<0.01.

Table 5 Differences of J-I-P test parameters under 5 different biofertilizer treatments of spinach leaf

The values presented

Treatments	J-I-P test parameters (relative unit)					
	M_0	PI_{ABS}	ABS/RC	TR_0/RC	ET_0/RC	DI_0/RC
CK	0.91±0.03ab	0.38±0.29c	2.20±0.08ab	1.37±0.06ab	0.46±0.04b	0.82±0.06a
T1	1.08±0.07a	1.24±0.21ab	2.57±0.03a	2.02±0.01ab	0.94±0.03a	0.55±0.02c
T2	1.00±0.02a	1.70±0.23a	2.59±0.03a	2.08±0.04a	1.07±0.01a	0.51±0.01b
T3	0.67±0.01ab	1.41±0.01bc	2.01±0.00b	1.43±0.02b	0.76±0.01ab	0.57±0.01ab
T4	0.83±001b	1.61±0.03bc	2.35±0.08ab	1.80±0.01ab	0.96±0.01ab	0.55±0.00bc
F value	7.66**	4.09*	2.88	3.97*	2.04	8.16**

Formatted Table

in each column of table have a mean ±standard deviation. The last part of table refers to the F value. * P<0.05, ** P<0.01.

Table 6 Differences of leaf chlorophyll fluorescence yield and efficiency under 5 different biofertilizer treatments of spinach

The values presented in each column of table have a mean ±standard

Treatments	Yield and efficiency			
	$\varphi P_0(F_v/F_m)$	φE_0	Ψ_0	φD_0
CK	0.62±0.01c	0.22±0.01ab	0.34±0.01ab	0.37±0.01ab
T1	0.78±0.01ab	0.37±0.01a	0.47±0.01a	0.21±0.00c
T2	0.80±0.00a	0.41±0.06b	0.52±0.05a	0.19±0.00bc
T3	0.72±0.0bc	0.38±0.03ab	0.53±0.02b	0.29±0.03a
T4	0.76±0.01ab	0.41±0.00ab	0.54±0.00ab	0.24±0.01bc
F value	6.03**	2.48	2.71	6.02**

Formatted Table

deviation. The last part of table refers to the F value. * $P<0.05$, ** $P<0.01$.

Table 7 Correlations between the leaf physiological characteristics and chlorophyll fluorescence parameters

Index	LW	LL	PH	SP	SC	Chl a	Chl b	Chl a+b	Chl a/b	MDA	WP
PI_{ABS}	0.75**	0.84**	0.92**	0.76**	0.81**	0.73**	0.55*	0.73**	0.68**	-0.71**	0.75**
φP_0	0.90**	0.89**	0.91**	0.89**	0.90**	0.93**	0.80**	0.93**	0.74**	-0.91**	0.90**
φE_0	0.58*	0.78**	0.89**	0.60*	0.65**	0.56*	0.38	0.55*	0.58*	-0.55*	0.58*
Ψ_0	0.74**	0.86**	0.95**	0.75**	0.79**	0.74**	0.56*	0.74**	0.68**	-0.72**	0.74**
φD_0	-0.90**	-0.89**	-0.91**	-0.89**	-0.90**	-0.93**	-0.80**	-0.93**	-0.74**	0.91**	-0.90**
ABS/RC	0.77**	0.51	0.40	0.78**	0.73**	0.83**	0.80**	0.84**	0.54*	-0.85**	0.78**
TR_0/RC	0.90**	0.74**	0.69**	0.90**	0.88**	0.94**	0.86**	0.96**	0.68**	-0.95**	0.90**
ET_0/RC	0.89**	0.88**	0.90**	0.91**	0.92**	0.91**	0.76**	0.92**	0.75**	-0.91**	0.90**

DI_0/RC	-0.80**	-0.90**	-0.97**	-0.78**	-0.82**	-0.80**	-0.65**	-0.81**	-0.70**	0.76**	-0.79**

* P<0.05** P<0.01. LL: Leaf length, LW: Leaf weight, PH: Plant height, SP: Soluble protein concentrations, SC: Soluble carbohydrate concentrations, MDA: malonaldehyde concentrations, Chl: chlorophyll, WP: Water potential.

Figure captions

Fig 1. Shape change of leaf chlorophyll fluorescence transient curve under 5 different biofertilizer treatments.

Each curve represents the average of three independent measurements. (A) Relative chlorophyll fluorescence intensity transient curves; (B) Relative variable fluorescence intensity transient curves (V_t); (C) Normalized the relative variable fluorescence intensity transient curves (ΔV_t); (D) Relative variable fluorescence intensity transient curves of the O-J segments (W_{OJ}); (E) Normalized the relative variable fluorescence intensity transient curves of the O-J segments (ΔW_{OJ}); (F)

Relative variable fluorescence intensity transient curves of the O-K segments (W_{OK}); (G) Normalized the relative variable fluorescence intensity transient curves of the O-K segments (ΔW_{OK})。 No fertilizer (CK), inoculations of *Bacillus subtilis* (*Bs*, T1), combination of *Bs* and *Bacillus mucilaginosus* (*Bs*+*Bm*, T2), *Bs* and *Bacillus amyloliquefaciens* (*Bs*+*Ba*, T3), and *Bm*+*Ba* (T4).

Fig 2. Light curve (LC) of quantum yield (QY) changes under 5 biofertilizer treatments.

Data are the means of three experiments. No fertilizer (CK), inoculations of *Bacillus subtilis* (*Bs*, T1), combination of *Bs* and *Bacillus mucilaginosus* (*Bs*+*Bm*, T2), *Bs* and *Bacillus amyloliquefaciens* (*Bs*+*Ba*, T3), and *Bm*+*Ba* (T4).

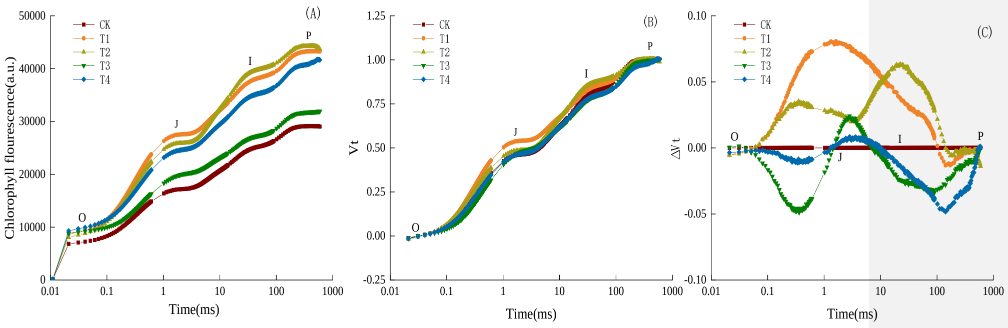

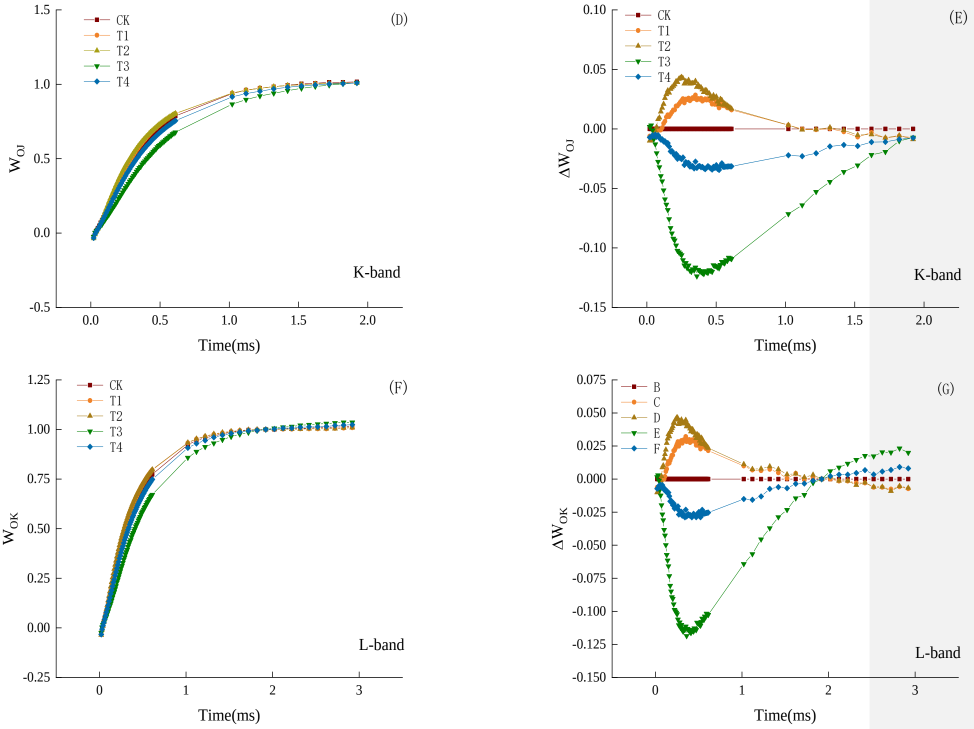

Fig 1Fig. 1 Shape change of leaf chlorophyll fluorescence transient curve under 5 different biofertilizer treatments. Each curve represents the average of three independent measurements. (A) Relative chlorophyll fluorescence intensity transient curves; (B) Relative variable fluorescence intensity transient curves (V_t); (C) Normalized the relative variable fluorescence intensity transient curves (ΔV_t); (D) Relative variable fluorescence intensity transient curves of the O-J segments (W_{OJ}); (E) Normalized the relative variable fluorescence intensity transient curves of the O-J segments (ΔW_{OJ}); (F) Relative variable fluorescence intensity transient curves of the O-K segments (W_{OK}); (G) Normalized the relative variable fluorescence intensity transient curves of the O-K segments (ΔW_{OK})。

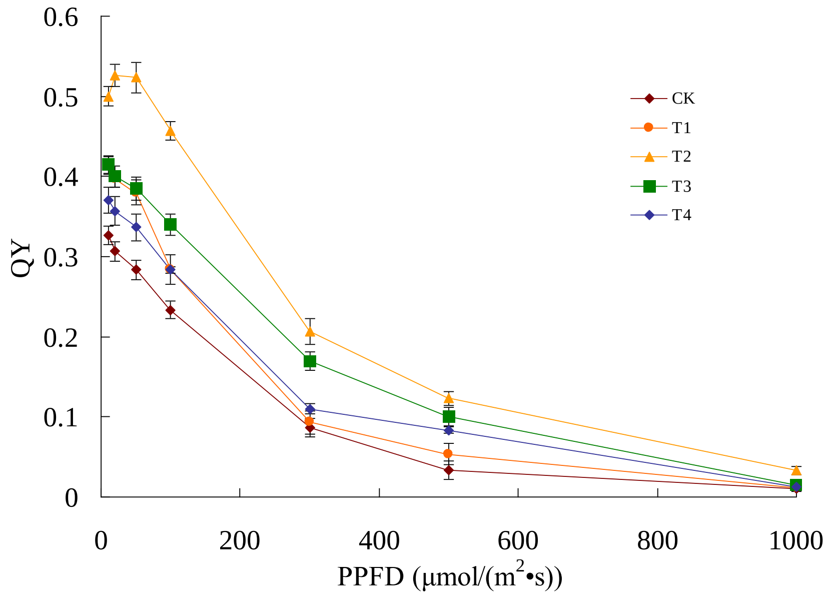

Fig 2

~~**Fig. 2 Light curve (LC) of quantum yield (QY) changes under 5 biofertilizer treatments. Data are the means of three experiments.**~~

Formatted: Indent: First line: 0 ch

Sep 4th, 2023

Editor Paula Katrina A. Maderazo
PLOS ONE

Dear Editor Paula Katrina A. Maderazo:

I have revised the manuscript # PONE-D-23-13632 based on editorial changes suggested by the reviewer1. The revisions are track-changed in the other part. I hope that the revisions have adequately addressed the editor's and reviewers' concerns.

Details of the revision conducted are in the following pages.

Yours sincerely
Beibei Zhang

Response to reviewers' comments:

Re: Manuscript # PONE-D-23-13632:
Review Comments to the Author

Reviewer #1: The authors have fulfilled the requirements for the manuscript, presented the research data, and the conclusions are substantiated.

The question - if there is an effect of fertilizers on chlorophyll fluorescence, then why is there no significant effect on the amount of chlorophyll? Then what is the reason for the change in fluorescence, it only with morphological changes in the leaf?

- **Accepted and Revised. In ms, the mean values were compared, and the difference in the amount of chlorophyll was also significant, but the F value was written incorrectly, we are so sorry about the mistake. When facing the external changes, the first change is the chlorophyll fluorescence, which could change the chlorophyll concentrations of the leaves and ultimately affects the morphological changes in the leaf.**

Reviewer #2: This was very well thought out experiment to test the effects of different inoculants on early spinach growth.

I had some issues reading and understanding parts of the manuscript. An additional edit for clarity would make it easier for readers to understand the work done and discussion presented. For example in the first paragraph of the discussion lines 246-250 "In this study, the inoculations of biofertilizer effectively improved the plant height, leaf length, and leaf weight of spinach, among which the growth status of plants with the inoculations of Bs and Bm was more dominant while which was similar to the results of Samia et al. [34] while the inoculation of Ba didn't have more effects.". Breaking long sentences like this up would improve how well this information is presented to the readers. Similarly changing awkward wording like "while which" and "didn't have more effects" would

provide a clearer point to the discussion. Improving sections like this throughout the manuscript would greatly improve the manuscript.

- **Accepted and Revised. We have revised in the ms.**

To also improve clarity, I would recommend that a more consistent representation of the treatments be decided upon. In parts of the manuscript the treatments are referred to as Bs/T1 or Bm/T2. For instance, in the introduction lines 129-131 identify the treatments as multiple identifiers (Bs, T1 or Bm T2), in the conclusion on line 328 the individual inoculants are identified (Bs and Bm), Fig 1 G has the graphs as (B,C,D,E, and F), , Table 2 lists the treatments as CK,T1,T2,T3,and T4. Deciding on a consistent representation for the treatments for the figures, tables, and text will make it easier for readers to better follow the effects of the treatments.

- **Accepted and Revised. Thank you for you comments, we have revised the representation making the ms more consistent and better understanding. In the lines 129-131, that was the material introductions. For the treatment introduce, it was on the experiment design part.**

In addition to the editing, I would recommend additional discussion of the impacts that the inoculants had on plant nitrogen status. It was mentioned in the introduction on lines 81-82 the impact that Bs has on changing the availability of nitrogen and phosphorus in the soil. Nitrogen was never mentioned again in the manuscript. Nitrogen is a major plant nutrient. Despite nitrogen status not being directly measured total chlorophyll could be used as a surrogate for plant nitrogen status. The impact the inoculants had on nitrogen would be a much better claim than the impact increased phosphorous had on ATP and NADPH production as was made in lines 264-268. With the data presented in table 3 the relationships between the various inoculants become much more apparent when look at the chlorophyll a+b data. When Bs and Bm were inoculated together (T2) the plants had more chlorophyll and likely nitrogen and phosphorous. Whereas when Bs or Bm were paired with Ba there was less chlorophyll and likely nitrogen and phosphorous. Then the

impacts on growth, leaf physiological indices and chlorophyll fluorescence response can be better framed around how the inoculant was impacting the plants. Presenting the results in the discussion in this context could better emphasize the potential impacts these inoculants have on early spinach growth and the importance or emphasis of species-specific inoculant and crop pairings.

- **Accepted and Revised. Thank you for your comments; we have discussed more in the ms.**

Lastly, I recommend that something be added to the manuscript that better frames the results obtained from an experiment growing spinach in a growth chamber at only 300 μmol/(m2·s). Spinach can be grown in full sunlight, 2000 μmol m-2·s-1, and discussing the impacts that the treatments in a growth chamber study with such low light on PS2 antenna could be misleading. Shade and low light treatments will inherently lead to increased PS2 antenna length. Therefore, given the experiment conducted, it would be difficult to conclude or suggest that the treatments impacted antenna length. They could have contributed to antenna length but cannot be certain of their impact.

- **Accepted and Revised. Thank you for your comments; we have discussed more in the discussion part. For the future experiment, we will design more light treatments to analyze the impact to spinach leaves.**

Overall, the manuscript in its current form is hard to understand (which took me time to read through it again and again). Thus, I strongly suggest for a language editor to go through the paper and present the data and findings in a more comprehensible yet intelligible form of standard English. The title needs to be drastically changed to a more compelling statement as it appears to be too “amateur with preliminary data”. Although when you read through the paper, it actually contains noteworthy data that would make a significant contribution to the field of agriculture. The experiments and other analysis were indeed performed to high technical standard although the statistical analysis needs

to be changed from Tukey’s test to Bonferroni test which is more appropriate based on its experimental design.

- **Accepted and Revised. We let a professional English speaker reviewed the paper and according to his comments we checked the entire article and revised.**
