# Supplementary material for: Effects of biofertilizers on the growth, leaf physiological indices and chlorophyll fluorescence response of spinach seedlings
Source: PLoS One. 2023 Dec 14;18(12):e0294349. doi: 10.1371/journal.pone.0294349 (PMC10721093; doi:10.1371/journal.pone.0294349)
Supplement: Attachment — Submitted filename: point to point Manuscript # PONE-D-22-05438.doc [file pone.0294349.s002.doc]

May 10^th^, 2023

PLOS ONE

Dear Editor Hilary Genn Givero:

I have revised the manuscript # PONE-D-22-05438 based on editorial changes suggested by the reviewer1. The revisions are track-changed in the other part. I hope that the revisions have adequately addressed the editor’s and reviewers’ concerns.

Details of the revision conducted are in the following pages.

Yours sincerely

Beibei Zhang

**Re: Manuscript # PONE-D-22-05438:**

**Review Comments to the Author**

Reviewer #1: The manuscript by Cheng et al. presents a compelling alternative to chemical fertilizers by using microbial fertilizers (3 Bacillus species) which promoted the growth of spinach seedlings and improved the adaptability of crops to soil by improving the effective phosphorus utilization rate. The authors further claim that the chlorophyll content, soluble protein, and physiological characteristics were improved. The study used three species of Bacillus: Bs: Bacillus subtilis, Ba:Bacillus amyloliquefaciens, Bm: Bacillus mucilaginosus. Based on their findings, Bs+Bm treatments resulted to improving the growth conditions of spinach seedlings exhibiting higher physiological index.

(1) However, there was no explanation on the non-efficacy of Ba whether it was Ba+Bs or Ba+Bm treatments yet the authors concluded that Bacillus (all 3 species) promoted plant growth.

- **Accepted and Revised. We have revised and added some explanations in the ms.**

(2) Moreover, in these kinds of experiments, it is essential that the garden soil used be fully described as this entails essential information that will support the applicability of microbial fertilizers, Bacillus sp. to be more precise.

- **Accepted and Revised. We have described the characteristics about the 3 Bacillus species in the ms.**

(3) Overall, the manuscript in its current form is hard to understand (which took me time to read through it again and again). Thus, I strongly suggest for a language editor to go through the paper and present the data and findings in a more comprehensible yet intelligible form of standard English.

- **Accepted and Revised. We let a professional English speaker reviewed the paper and according to his comments we checked the entire article and revised.**

The title needs to be drastically changed to a more compelling statement as it appears to be too “amateur with preliminary data”.

- **Revised.**

Although when you read through the paper, it actually contains noteworthy data that would make a significant contribution to the field of agriculture.

- **Accepted.**

The experiments and other analysis were indeed performed to high technical standard although the statistical analysis needs to be changed from Tukey’s test to Bonferroni test which is more appropriate based on its experimental design.

- **Accepted and Revised.**
