# Supplementary material for: Effects of biofertilizers on the growth, leaf physiological indices and chlorophyll fluorescence response of spinach seedlings
Source: PLoS One. 2023 Dec 14;18(12):e0294349. doi: 10.1371/journal.pone.0294349 (PMC10721093; doi:10.1371/journal.pone.0294349)
Supplement: Attachment — Submitted filename: response letter-2023-9-4.doc [file pone.0294349.s003.doc]

Sep 4^th^, 2023

Editor Paula Katrina A. Maderazo

PLOS ONE

Dear Editor Paula Katrina A. Maderazo:

I have revised the manuscript # PONE-D-23-13632 based on editorial changes suggested by the reviewer1. The revisions are track-changed in the other part. I hope that the revisions have adequately addressed the editor’s and reviewers’ concerns.

Details of the revision conducted are in the following pages.

Yours sincerely

Beibei Zhang

**Response to reviewers’ comments:**

**Re: Manuscript # PONE-D-23-13632:**

**Review Comments to the Author**

Reviewer #1: The authors have fulfilled the requirements for the manuscript, presented the research data, and the conclusions are substantiated.

The question - if there is an effect of fertilizers on chlorophyll fluorescence, then why is there no significant effect on the amount of chlorophyll? Then what is the reason for the change in fluorescence, it only with morphological changes in the leaf?

- **Accepted and Revised. In ms, the mean values were compared, and the difference in the amount of chlorophyll was also significant, but the F value was written incorrectly, we are so sorry about the mistake. When facing the external changes, the first change is the chlorophyll fluorescence, which could change the chlorophyll concentrations of the leaves and ultimately affects the morphological changes in the leaf.**

Reviewer #2: This was very well thought out experiment to test the effects of different inoculants on early spinach growth.

I had some issues reading and understanding parts of the manuscript. An additional edit for clarity would make it easier for readers to understand the work done and discussion presented. For example in the first paragraph of the discussion lines 246-250 “In this study, the inoculations of biofertilizer effectively improved the plant height, leaf length, and leaf weight of spinach, among which the growth status of plants with the inoculations of Bs and Bm was more dominant while which was similar to the results of Samia et al. [34] while the inoculation of Ba didn’t have more effects.”. Breaking long sentences like this up would improve how well this information is presented to the readers. Similarly changing awkward wording like “while which” and “didn’t have more effects” would provide a clearer point to the discussion. Improving sections like this throughout the manuscript would greatly improve the manuscript.

- **Accepted and Revised. We have revised in the ms.**

To also improve clarity, I would recommend that a more consistent representation of the treatments be decided upon. In parts of the manuscript the treatments are referred to as Bs/T1 or Bm/T2. For instance, in the introduction lines 129-131 identify the treatments as multiple identifiers (Bs, T1 or Bm T2), in the conclusion on line 328 the individual inoculants are identified (Bs and Bm), Fig 1 G has the graphs as (B,C,D,E, and F), , Table 2 lists the treatments as CK,T1,T2,T3,and T4. Deciding on a consistent representation for the treatments for the figures, tables, and text will make it easier for readers to better follow the effects of the treatments.

- **Accepted and Revised. Thank you for you comments, we have revised the representation making the ms more consistent and better understanding.** **In the lines 129-131, that was the material introductions. For the treatment introduce, it was on the experiment design part.**

In addition to the editing, I would recommend additional discussion of the impacts that the inoculants had on plant nitrogen status. It was mentioned in the introduction on lines 81-82 the impact that Bs has on changing the availability of nitrogen and phosphorus in the soil. Nitrogen was never mentioned again in the manuscript. Nitrogen is a major plant nutrient. Despite nitrogen status not being directly measured total chlorophyll could be used as a surrogate for plant nitrogen status. The impact the inoculants had on nitrogen would be a much better claim than the impact increased phosphorous had on ATP and NADPH production as was made in lines 264-268. With the data presented in table 3 the relationships between the various inoculants become much more apparent when look at the chlorophyll a+b data. When Bs and Bm were inoculated together (T2) the plants had more chlorophyll and likely nitrogen and phosphorous. Whereas when Bs or Bm were paired with Ba there was less chlorophyll and likely nitrogen and phosphorous. Then the impacts on growth, leaf physiological indices and chlorophyll fluorescence response can be better framed around how the inoculant was impacting the plants. Presenting the results in the discussion in this context could better emphasize the potential impacts these inoculants have on early spinach growth and the importance or emphasis of species-specific inoculant and crop pairings.

- **Accepted and Revised. Thank you for your comments; we have discussed more in the ms.**

Lastly, I recommend that something be added to the manuscript that better frames the results obtained from an experiment growing spinach in a growth chamber at only 300 μmol/(m2·s). Spinach can be grown in full sunlight, 2000 μmol m-2·s-1, and discussing the impacts that the treatments in a growth chamber study with such low light on PS2 antenna could be misleading. Shade and low light treatments will inherently lead to increased PS2 antenna length. Therefore, given the experiment conducted, it would be difficult to conclude or suggest that the treatments impacted antenna length. They could have contributed to antenna length but cannot be certain of their impact.

- **Accepted and Revised. Thank you for your comments; we have discussed more in the discussion part. For the future experiment, we will design more light treatments to analyze the impact to spinach leaves.**

Overall, the manuscript in its current form is hard to understand (which took me time to read through it again and again). Thus, I strongly suggest for a language editor to go through the paper and present the data and findings in a more comprehensible yet intelligible form of standard English. The title needs to be drastically changed to a more compelling statement as it appears to be too “amateur with preliminary data”. Although when you read through the paper, it actually contains noteworthy data that would make a significant contribution to the field of agriculture. The experiments and other analysis were indeed performed to high technical standard although the statistical analysis needs to be changed from Tukey’s test to Bonferroni test which is more appropriate based on its experimental design.

- **Accepted and Revised. We let a professional English speaker reviewed the paper and according to his comments we checked the entire article and revised.**
