# Supplementary material for: Effects of biofertilizers on the growth, leaf physiological indices and chlorophyll fluorescence response of spinach seedlings
Source: PLoS One. 2023 Dec 14;18(12):e0294349. doi: 10.1371/journal.pone.0294349 (PMC10721093; doi:10.1371/journal.pone.0294349)
Supplement: Attachment — Submitted filename: Review_comment_to_authors_PONE.docx [file pone.0294349.s005.docx]

**Manuscript Number PONE-D-23-13632 "Effects of biofertilizers on the growth, leaf physiological Indices and chlorophyll fluorescence response of spinach seedlings"**

Review Comments to the Author

The manuscript is of interest to the journal however it presents many problems related to the interpretation of the results obtained.

In the description of the results the authors never take into account the results of the statistical analysis applied to the data obtained (see comments in the text) and therefore the discussion is also misleading

In addition, some sentences do not make any sense and there is no consequential and logical discussion.

Even the interpretation of some parameters obtained with the determination of chlorophyll fluorescence reveals a lack of knowledge of the latter
