# Supplementary material for: Effects of biofertilizers on the growth, leaf physiological indices and chlorophyll fluorescence response of spinach seedlings
Source: PLoS One. 2023 Dec 14;18(12):e0294349. doi: 10.1371/journal.pone.0294349 (PMC10721093; doi:10.1371/journal.pone.0294349)
Supplement: Attachment — Submitted filename: response letter-2023-10-30.doc [file pone.0294349.s006.doc]

Oct 30^th^, 2023

Editor Mayank Gururani

PLOS ONE

Dear Editor Mayank Gururani:

I have revised the manuscript # PONE-D-23-13632R1 based on editorial changes suggested by the reviewers. The revisions are track-changed in the other part. I hope that the revisions have adequately addressed the editor’s and reviewers’ concerns.

Details of the revision conducted are in the following pages.

Yours sincerely

Beibei Zhang

**Response to reviewers’ comments:**

**Re: Manuscript # PONE-D-23-13632R1:**

**Review Comments to the Author**

**Reviewer #4**: The manuscript is of interest to the journal however it presents many problems related to the interpretation of the results obtained.

In the description of the results the authors never take into account the results of the statistical analysis applied to the data obtained (see comments in the text) and therefore the discussion is also misleading.

- **Revised. Thank you for your comments; we have discussed more in the ms and some details were shown in the following pages.**

In addition, some sentences do not make any sense and there is no consequential and logical discussion.

- **Revised. Thank you for your comments; we have discussed more in the ms.**

Even the interpretation of some parameters obtained with the determination of chlorophyll fluorescence reveals a lack of knowledge of the latter.

- **Revised. Thank you for your comments; we have discussed more in the ms.**

Line 60	The plant species?

- **Revised.**

Line 86	‘covert’ changed to ‘coverts’.

- **Revised.**

Line 89	‘performances’ changed to ‘indicators’.

- **Revised.**

Line 98,99	‘significant’ changed to ‘a significant’? ‘Verticillium’ should be italic.

- **Revised.**

Line 127-131 when was applied?

- **Revised. It was applied at March 10th, 2021.**

Line 181 ‘and other growth parameters’ which parameters?

- **Revised. Sorry about that, and here we didn’t analyze the other growth parameters.**

Line 183 ‘was maximum under the T2 treatment’ The sentence it is not true because T2 is similar to T1.

- **Revised. From the original data we can see actually it was maximum under T2 and for the statically analysis they didn’t have significant differences.**

Line 185	‘And the T2 treatment had the greatest impact on the leaf physiological responses.’ This sentence it is not true for all physiological parameters.

- **Revised. From the original data we can see actually it was maximum under T2 and for the statically analysis they didn’t have significant differences.**

Lime 186	‘Compared with the F value, the difference in soluble sugar (SC) was the highest (F=118.35; P<0.01).’ this sentence has not sense.

- **Revised.**

Line 192 Table 3 as it is possible that 0.58 is ab and 0.6 is b for Chl b.

- **Revised. Sorry about that, here we wrote the wrong letter.**

Line 201	‘value of the T2 treatment was the highest’ this sentence it is not true.

- **Revised. The F_0_ value under T2 was 10013, it was the highest. Sorry that we labelled the wrong letter in Table 4 and revised.**

Line 205	‘of the F_v_/F_0_ value, the T2 treatment remained the largest’ this sentence it is not true.

- **Revised. The Fv/F0 value under T2 was 4.08, which was the highest. We labelled the wrong letter in Table 4 and revised..**

Line 206	‘Compared with the F value, the difference of F0 was the largest (F=112.57; P < 0.01).’ this sentence has not sense.

- **Revised.**

Line 217-218	‘treatment had the greatest effect on the specific activity of each reaction center (RC), and there was a significant difference between the TR_0_/RC and DI_0_/RC.’ this sentence it is not true.

- **Revised.**

Line 220-222 ‘The quantum yield (φP_0_), efficiency (φE_0_), and ψ_0_ values were highest under the T2 treatment, and with the exception of ψ_0_, there were significant differences (Table 6) (P<0.01).’ this sentence it is not true.

- **Revised.**

Line 224-225	Table 6.

- **Revised.**

Line 233-234	‘the fluorescence value of the T2 treatment was the largest, which signified that it had a greater effect on the photosynthetic chemical rate of the leaves.’ this sentence it is not true.

- **Revised.**

Line 235-236	‘As shown in Fig 1(B), the difference of relative variable fluorescence (V_t_) at point J (2 ms) was the largest.’ This is not evident in Fig 1B.

- **Revised. We analyzed the V_t_ data at point J, the ANOVA showed it had the highest differences (F=2354.87).**

Line 275-276	‘The growth status of plants with the inoculations of *Bs* or *Bm* was more dominant and it was similar to the results of Samia et al. [34].’ this sentence it is not true.

- **Revised.**

Line 281-282 ‘inoculated together, the leaf physiological indices of spinach seedling (SP, SC, Chl a, Chl b, Chl a+b, Chl a/b, and WP) were significantly enhanced.’ This is not true for Chlb and Chla/b.

- **We have revised the letters in Table 3. For Chlb and Chla/b, they were also significantly enhanced.**

Line 284-285	‘The application of phosphate fertilizer can promote the synthesis of chlorophyll in leaves [37].’ What means this sentence?

- **Revised. At first, we want to use the fertilizer adding can improve the synthesis of chlorophyll in leaves to explain our data.**

Line 291-293	‘Thus promoting the synthesis of ATP (Adenosine triphosphate) and NADPH (nicotinamide adenine dinucleotide phosphate) in leaves then promoting the growth.’ This sentence has no sense.

- **Revised.**

Line 294-295	‘phytohormone biosynthesis pathways; modulate ethylene levels in plants and the launch of host plants’ systemic tolerance.’ It is not possible to hypothesize because author didn’t determine the molecules.

- **Revised. We cited the relevant research results from other studies.**

Line 302-303	‘the concentration of MDA in leaves treated with biofertilizer was decreased which mean the plant was in a suitable environment.’ Why MDA amount is so high in control?

- **Maybe because in control it was under a stress condition and the MDA was higher, while after biofertilizer adding it was under a suitable condition and the MDA amount was lower.**
